# Effectiveness of CoronaVac in a pioneer risk-based allocation clinical trial during the COVID-19 pandemic

**DOI:** 10.1371/journal.pone.0351566

**Published:** 2026-06-22

**Authors:** Alex Martins, Mario Felipe Bosco Santos, Joabi Nascimento, Fabíola Mendonça da Silva Chui, Maria Gabriela Almeida Rodrigues, Talita Bastos, Sonia Lemos, Eduardo Honorato, Márcio Cortez, Mariana Simão Xavier, Erika Oliveira Gomes, Rebeca Linhares Abreu-Netto, Salete Fernandes, Alexandre Vilhena Silva-Neto, André Sachett, Bernardo Maia Silva, Gabriel Santos Mouta, Jady Shayenne Mota Cordeiro, Djane Clarys Baía-da-Silva, José Diego Brito-Sousa, Karina Pinheiro Pessoa, Maurício Lacerda Nogueira, Ricardo Palacios, Wuelton Monteiro, Felipe Naveca, Vanderson Sampaio, Fernando Almeida-Val, Gisely Melo, Otavio Ranzani, Maria Paula Mourão, Marcus Lacerda

**Affiliations:** 1 Fundação de Medicina Tropical Dr Heitor Vieira Dourado, Manaus, Brazil; 2 Universidade do Estado do Amazonas, Manaus, Brazil; 3 Instituto Nacional de Infectologia Evandro Chagas-Fiocruz, Rio de Janeiro, Brazil; 4 Instituto Nacional de Pesquisas da Amazônia - INPA, Manaus, Brazil; 5 Instituto Leônidas & Maria Deane, Fiocruz, Manaus, Brazil; 6 Universidade Nilton Lins, Manaus, Brazil; 7 Faculdade de Medicina de São José do Rio Preto, São Paulo, Brazil; 8 Instituto Butantan, São Paulo, Brazil; 9 Duke Global Health Institute, Duke University, Durham, United States of America; 10 Fundação de Vigilância em Saúde Dra Rosemary Costa Pinto, Manaus, Brazil; 11 Instituto Todos pela Saúde, São Paulo, Brazil; 12 ISGlobal, Barcelona, Spain; 13 University of Texas Medical Branch, Galveston, United States of America; College of Medical Sciences, NEPAL

## Abstract

CoronaVac, an inactivated SARS-CoV-2 vaccine, was one of the first deployed during the COVID-19 pandemic. Given the limited vaccine availability and the urgent need to assess effectiveness in target populations, a risk-based allocation clinical trial was designed to generate evidence under the ethical and logistical constraints at the beginning of the COVID-19 vaccination. In Manaus, Brazil, participants working in public service (education and public safety) aged 18–49 years were assessed regarding the risk of severe COVID-19 disease. Participants with one or more comorbidities, who were considered at higher risk of severe COVID-19 outcomes if infected, were allocated to early vaccination, while participants without comorbidities were enrolled as an unvaccinated comparison group. Blood samples were collected before each vaccine dose (D0 and D28) and during in-person follow-up visits (D90 and D180). Additional information was obtained through phone calls. Clinical cases of COVID-19, hospitalizations, deaths, and antibody titrations were the evaluated endpoints, considered after the second week following the second dose of the vaccine. A total of 6,226 participants were included: 1,139 in the low-risk group, and 5,087 in the high-risk vaccinated group. COVID-19 incidence was statistically significantly lower (*p* < 0.001) in the vaccinated group at D28 (5.2% vs. 40.9%) and D90 (10.3% vs 31.6%). Hospitalization and death rates were low, with no difference observed between the groups. There was a decline in highly reactive titers at D180 in the vaccinated group. This pioneer risk-based allocation clinical trial provided evidence that CoronaVac reduced the risk of severe COVID-19 outcomes among individuals with comorbidities, effectively aligning their risk with that of lower-risk, unvaccinated individuals. Beyond its clinical implications, the study underscores the importance of adaptive, real-world research designs in rapidly generating actionable evidence in response to emerging public health threats.

ClinicalTrials.gov Registration: NCT04789356

## Introduction

The rapid development of vaccines against SARS-CoV-2 played a central role in reducing the global burden of the COVID-19 pandemic [1,2], with substantial early reductions in COVID-19 symptomatic and severe outcome rates following vaccination [3]. High vaccination rates and naturally acquired immunity have helped reduce cases and deaths from COVID-19, culminating in the end of the Public Health Emergency of International Concern in 2023 [4,5].

The CoronaVac inactivated whole-virion SARS-CoV-2 vaccine (Sinovac) was one of the first COVID-19 vaccines extensively used globally, particularly in low-and-middle income countries [4]. Clinical trials and real-world studies have demonstrated that the vaccine effectively reduced the severity of the disease and mortality, with minimal adverse events reported [4–7]. Furthermore, this vaccine has shown efficacy against various SARS-CoV-2 variants, including the Gamma variant first identified in samples from Manaus, Brazil [8–10].

CoronaVac was among the first vaccines approved and distributed on a large scale in Brazil in 2021. In Manaus, where COVID-19 cases and death rates exceeded those of the rest of the country during the second wave caused by the Gamma [10], the limited vaccine availability necessitated targeted campaigns to ensure protection of the most vulnerable populations [11,12]. Brazilian national guidelines began vaccinating the older population and healthcare professionals first. This targeted approach aimed to alleviate the burden on healthcare systems and protect those at higher risk of severe outcomes from the virus [12,13]. Due to the limited availability of more vaccines, younger groups, even those living with comorbidities, were not initially prioritized for vaccination at the beginning of 2021.

In 1996, Finkelstein et al [14] proposed allocating experimental interventions according to the individual participant’s risk to avoid the ethical challenge of potentially denying the best opportunity to those in the highest need. A control group, deliberately selected to have a lower risk, would also be concurrent; therefore, it would differ only in terms of risk factors and the intervention. The idea behind it is to demonstrate that the intervention can decrease the risk of an adverse outcome in those more likely to have one to the level of those with a better prognosis. Therefore, a clear identification of risk factors and an understanding of ethical constraints are required to consider the proposed risk-based allocation trial design. Nonetheless, no clinical study has been published to date using this design.

Given the resource-limited setting at the start of the COVID-19 vaccination campaign, we proposed a risk-based allocation clinical trial anticipating the use of CoronaVac in patients aged 18–49 with comorbidities, a population known to have increased COVID-19 mortality, but not initially targeted according to the Brazilian Ministry of Health Guidelines. We hypothesized that COVID-19-associated mortality in this group would be similar to that of a same age group without comorbidities and not vaccinated.

## Methods

### Study design

This study was a risk-based allocation clinical trial with non-equivalent groups, conducted in Manaus, Brazil, during the massive circulation of the Gamma variant of SARS-CoV-2, to assess the effectiveness of the inactivated, adsorbed COVID-19 vaccine CoronaVac, the same used in the country at the time. The study team evaluated individuals aged 18–49 years who worked in education or public safety sectors in Manaus, considered to be more exposed to the infection than the general population. All participants were informed that they would be followed up during the study period; however, only those identified as having a higher risk of severe COVID-19 outcomes, as defined by the National Plan for the Implementation of Vaccination against COVID-19, received early access to vaccination within the context of this project. In contrast, low-risk participants (those without comorbidities and hence a lower risk of severe COVID-19 outcomes if infected) were not prioritized for early vaccination and served as a comparison group. COVACMANAUS trial was registered at ClinicalTrials.gov (NCT04789356) and was approved by the Brazilian National Research Ethics Commission (CAAE 44076721.5.0000.0005). All participants provided informed written consent. The authors confirm that all ongoing and related trials for this drug/intervention are registered.

### Participants

During the study, vaccination in Manaus was limited to health workers and those over 50 years of age, due to constrained supply. Between March 18 and April 8, 2021, education and public safety professionals were invited to participate based on their occupational risk. Candidates for vaccine anticipation were pre-screened virtually (online, in partnership with the local government secretariats) and scheduled for in-person eligibility assessment. Inclusion criteria were: (a) age 18–49 years; (b) availability for study 180-day follow-up; and (c) presence of at least one comorbidity listed in the National Immunization Plan (PNI) (S1 Table) [15]. Participants over 50 were not included, as they had already been included in the national vaccine rollout campaign.

Exclusion criteria included: (a) prior COVID-19 vaccination; (b) diagnosis of COVID-19 in the past 28 days; (c) history of severe allergic reaction or anaphylaxis to any component of the study vaccine; (d) self-reported fever within the prior 72 hours (inclusion could be postponed until 72 hours had passed without fever); (e) receipt of a live attenuated vaccine in the previous 28 days or an inactivated vaccine in the previous 14 days, or having immunization scheduled within 28 days of enrollment; (f) any condition judged by the investigator as compromising safety or protocol compliance; and (g) pregnancy or breastfeeding. Only permanent residents of Manaus were eligible due to the study’s longitudinal design. Enrollment for both low-risk and high-risk groups occurred simultaneously.

### Intervention

A single research batch of CoronaVac (Sinovac Biotech, China) was used in the study, with mono-dose vials provided as a donation by the Butantan Institute in Brazil. Each dose was stored in individual, ready-to-use research vials at the state regulatory institution, *Fundação de Vigilância em Saúde Dra. Rosemary Costa Pinto,* in Manaus, was transported daily to the research site and kept at 2–8 °C as per the manufacturer’s instructions. The vaccine (3 μg in 0.5 mL) was administered in two doses, 28 days (±7 days) apart, via intramuscular injection.

### Procedures and follow-up

After fulfilling the eligibility criteria, demographic information, anthropometrics, self-reported pre-existing medical conditions, and contact information were collected. Blood samples were collected before each vaccine dose and during in-person follow-up visits. Solicited and unsolicited follow-up contacts (by phone, electronic means, or in-person) were made within the first 7 days after each vaccine dose to monitor for adverse events and COVID-19 symptoms [16]. In-person visits occurred at D0 (first dose), D28 (second dose), D90, and D180, with a follow-up phone call on D120.

Nasal and oropharyngeal swab samples were collected from symptomatic participants. For the detection of SARS-CoV-2, the Panbio COVID-19 Antigen rapid test device assay (Abbott, USA) on nasopharyngeal samples was performed. According to the manufacturer’s recommendations, swab specimens (nasopharyngeal or oropharyngeal swabs) were used to extract viral RNA with the QIAamp Viral RNA Mini Kit (QIAGEN, Germany). Subsequently, they were tested for SARS-CoV-2 using real-time RT–PCR as a routine diagnostic for COVID-19 using any of the following commercial assays: SARS-CoV-2 (E/RP) (Biomanguinhos), Allplex 2019-nCoV Assay (Seegene), or an in-house protocol following US Centers for Disease Control and Prevention (CDC) guidelines (https://www.fda.gov/media/134922/download). For all assays, specimens were considered positive if both viral targets, N1 and N2, showed cycle thresholds (CT) of 40.0 or lower. IgG antibodies against the Spike protein were measured by Elecsys Anti-SARS-CoV-2 S commercial kits (Roche, USA). The procedure was performed per the manufacturer’s instructions [17]. The samples were categorized as reactive (>0.8 U/mL) or highly reactive (≥250 U/mL) for antibodies targeting the receptor-binding domain (RBD) of the Spike protein. No further dilutions were performed after 250 U/mL.

Clinical and laboratory data from all-cause hospitalizations were also retrieved, if applicable. Additionally, a probabilistic record linkage was conducted monthly between the cohort and SIVEP-Gripe (the national surveillance database for severe acute respiratory infections) and SIM (the official Mortality Information System in Brazil) to identify individuals with hospitalizations, COVID-19 diagnoses, or deaths that were not captured through active follow-up. All data were collected in REDCap electronic forms.

### Sample size

Using a 0.41% and 1.03% incidence of hospitalization in the general population and in patients with comorbidities, respectively, according to the Brazilian Institute of Geography and Statistics (IBGE) and *SIVEP-Gripe*, at the time of the study design, one-tailed alpha of 5%, power of 90%, annual loss rate of 30%, the study required 10,156 participants to be included in the 1:1 ratio (5,078 at high-risk individuals for severe COVID-19 to receive the vaccine and 5,078 at low-risk individuals who would not receive the vaccine). Although we had planned a 1:1 allocation ratio between groups, the Ministry of Health (MoH) expanded vaccine availability to the study target population progressively after February 2021, allowing those without comorbidities in the study age range to be vaccinated, decreasing the non-vaccination group, resulting in a final 4:1 ratio [18].

### Outcomes

The primary outcome was the incidence of moderate and severe clinical cases of COVID-19 leading to hospitalization (grade 4 or higher according to the WHO clinical progression scale), after the second week following the second dose of the vaccine. Other outcomes included the titration of IgG anti-RBD antibodies against SARS-CoV-2, the presence of virologically confirmed clinical cases of COVID-19, and mortality associated with COVID-19 at days 28, 90, 120, and 180. The control group was followed up until they were vaccinated routinely in the public health system.

### Statistical analysis

For baseline demographic and clinical data, continuous variables are reported as means with standard deviations (and ranges), and categorical variables as counts and percentages. Student’s t or Mann-Whitney tests were used to compare continuous variables, and chi-squared or Fisher’s test was used to assess differences between proportions. The proportions were compared between groups at the evaluated time intervals using the chi-square test for equality of proportions, with normal approximation and Yates’ continuity correction. Probabilistic data linkage was performed using the package fastLink (version 0.6.0) in R software (version 4.1.0). Statistical analyses were performed using Stata v17.

### Role of the funding source

The funders had no role in study design, data collection and analysis, decision to publish, or preparation of the manuscript.

## Results

**Fig 1:**
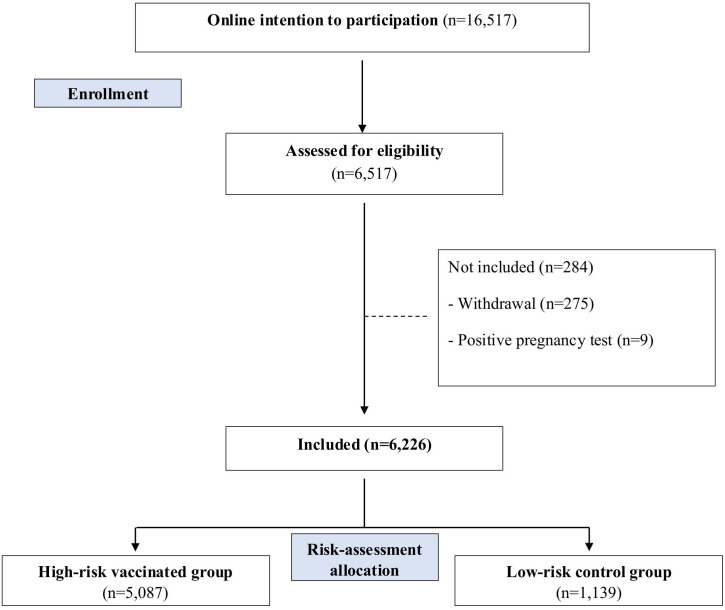
Flowchart of participant inclusion. Recruitment and screening occurred simultaneously for both groups.

Between March 18 and April 8, 2021, a total of 16,517 participants expressed their intention to participate in the study via online screening. After eligibility screening, 6,226 participants were included: 1,139 (18%) in the low-risk control group, and 5,087 (72%) in the High-risk vaccinated group (Fig 1).

Table 1 summarizes the baseline data of the participants. In the high-risk vaccinated group, the most common comorbidities were obesity (81.1%), systemic arterial hypertension (20.1%), and diabetes (10.5%). Of 5,087 vaccinated participants, sixteen (0.3%) did not receive the second dose.

**Table 1:** Baseline characteristics of study participants.

Variable	Low-risk control group	High-risk vaccinated group	Total
n = 1,139	n = 5,087	N = 6,226
Sex, female, %	791 (69.4)	3,046 (59.9)	3,837 (61.6)
Age, years, Median (IQR)	38 (32-43)	40 (35-45)	40 (34-45)
Race, %			
White	243 (21.3)	1,162 (22.8)	1,405 (22.6)
Black	79 (6.9)	403 (7.9)	482 (7.7)
Admixed*	782 (68.7)	3,383 (66.5)	4,165 (66.9)
Asian	21 (1.8)	82 (1.6)	103 (1.6)
Indigenous	11 (1.0)	43 (0.8)	54 (0.9)
Chronic comorbidities, n/N (%)			
Obesity	–	4,128 (81.1)	–
Systemic arterial hypertension	–	1023 (20.1)	–
Diabetes	–	532 (10.5)	–
Immunosuppressed	–	241 (4.7)	–
Severe chronic lung disease	–	233 (4.6)	–
Cardiovascular diseases	–	84 (1.6)	–
Cerebrovascular disease	–	12(0.2)	–
Chronic kidney disease	–	12 (0.2)	–
Sickle cell anemia	–	3 (0.1)	–
Hepatic cirrhosis	–	1 (<0.1)	–
Positive anti-Spike IgG in D0, %	674/1,117 (60.3)	3,012/4,839 (62.2)	3,686/5,956 (61.9)

*Admixed: mixed genetic ancestry, commonly Indigenous American, European, and African in the Brazilian population.

At baseline (D0), 61.9% of participants had reactive serology (greater than 0.8 U/mL): 60.3% in the unvaccinated comparison group and 62.2% in the vaccinated group (Table 1). The latter also exhibited highly reactive serology (≥250 U/mL) compared to the low-risk control group at baseline and D90, with a decline in highly reactive titers observed at D180 (Table 2). There was a significant reduction in COVID-19 infections among vaccinated participants during the first 28 and 90 days of follow-up, with a continued, albeit smaller and not statistically significant, reduction at 120 and 180 days (Table 2). Vaccinated high-risk individuals had similar severe outcomes (hospitalization and death) to unvaccinated low-risk individuals at all points analyzed (Table 2).

**Table 2:** COVID-19 endpoints and serodetection among participants during follow-up.

	Total	Low-risk control group	High-risk vaccinated group	*p*		
	N = 6,222	n = 1,135	n = 5,087		95%CI	
**D0 to D28**						
COVID-19, %	306/5,175 (5.9)	43/105 (40.9)	263/5,070 (5.2)	<0.001	−0.46	−0.26
Hospitalization by COVID-19, %	7/6,155 (0.1)	0/1,096 (0.0)	7/5,059 (0.1)	0.46	−0.0002	0.003
Death by COVID-19, %	0/6,192 (0.0)	0/1,113 (0.0)	0/5,079 (0.0)	–		
Reactive serology in D0 (>0.8 U/mL), %	3,684/5,953 (61.9)	672/1,114 (60.3)	3,012/4,839 (62.2)	0.25	−0.01	0.05
Reactive serology in D0 (≥250 U/mL), %	1,659/5,953 (27.9)	199/1,114 (17.9)	1,460/4,839 (30.2)	<0.001	0.10	0.15
Reactive serology in D28 (>0.8 U/mL), %	4,600/5,031 (91.4)	–	4,600/5,031 (91.4)	–		
Reactive serology in D28 (≥250 U/mL), %	3,005/5,031 (59.7)	–	3,005/5,031 (59.7)	–		
**D0 to D90**		**371**	**5,087**			
COVID-19, %	536/5,111 (10.5)	12/38 (31.6)	524/5,073 (10.3)	<0.001	−0.37	−0.05
Hospitalization by COVID-19, %	10/5,344 (0.2)	1/343 (0.3)	9/5,001 (0.2)	1.00	−0.008	0.006
Death by COVID-19, %	0/5,428 (0.0)	0/349 (0.0)	0/5,079 (0.0)	–		
Reactive serology in D90 (>0.8 U/mL), %	4,610/4,624 (99.7)	10/13 (76.9)	4,600/4,611 (99.8)	<0.001	0.006	0.03
Reactive serology in D90 (≥250 U/mL), %	2,789/4,624 (60.3)	5/13 (38.5)	2,784/4,611 (60.4)	0.48	−0.02	0.05
D0 to D120*		**177**	**5,087**			
COVID-19, %	614/5,085 (12.1)	2/10 (20.0)	612/5,075 (12.1)	0.78	−0.38	0.22
Hospitalization by COVID-19, %	12/5,139 (0.2)	0/155 (0.0)	12/4,984 (0.2)	1.00	−0.001	0.006
Death by COVID-19, %	1/5,235 (0.0)	0/155 (0.0)	1/5,080 (0.0)	1.00	−0.0004	0.0008
Reactive serology in D180 (>0.8 U/mL), %	N/A	N/A	N/A			
Reactive serology in D180 (≥250 U/mL), %	N/A	N/A	N/A			
**D0 to D180**		**168**	**5,087**			
COVID-19, %	667/5,087 (13.1)	3/10 (30.0)	664/5,077 (13.1)	0.26	−0.50	0.17
Hospitalization by COVID-19, %	12/5,083 (0.2)	0/146 (0.0)	12/4,937 (0.2)	1.00	−0.001	0.006
Death by COVID-19, %	1/5,226 (0.0)	0/146 (0.0)	1/5,080 (0.0)	1.00	−0.0004	0.0008
Reactive serology in D180 (>0.8 U/mL), %	4,552/4,565 (99.7)	–	4,552/4,565 (99.7)	–		
Reactive serology in D180 (≥250 U/mL), %	2,185/4,565 (47.8)	–	2,185/4,565 (47.8)	–		

* *Phone call visit; N/A, not available. COVID-19 is presented as n/N (%), where n is the number of participants with confirmed COVID-19 and N is the number of symptomatic participants screened/tested for COVID-19 during the specified time interval.*

Hospitalization rates were low in both groups, with 0.14–0.24% observed among high-risk vaccinated individuals across all follow-up periods. In comparison, low-risk unvaccinated individuals had hospitalization rates ranging from 0.0% to 0.29%, though the number of participants in this group decreased substantially over time. The absolute risk reduction between groups was modest (up to 0.15%), yielding an estimated number needed to treat (NNT) of approximately 667 to prevent one additional COVID-19-related hospitalization. This NNT reflects a pragmatic estimate of vaccine effectiveness in reducing hospitalizations among high-risk individuals in a real-world setting. However, given the small number of hospitalization events and the lack of statistical significance in group comparisons, this estimate should be interpreted cautiously. Furthermore, the initially calculated sample size (n = 10,156) was not reached, and the control group declined substantially during follow-up (from 1,139 to fewer than 150 participants by day 180), limiting the power of comparative analyses. The high baseline seroprevalence (61.9%) in both groups may also have attenuated the measurable effect of vaccination by conferring partial immunity at study entry.

## Discussion

Several authors have discussed theoretical and mathematical models for allocating interventions based on risk [19]. Still, this study is the first reported in the literature to be prospectively designed as a risk-based allocation clinical trial. In the original proposal, Finkelstein et al [14] acknowledged that evidence generation to prove the efficacy of an intervention can eventually utilize other controls, such as historical controls, rather than the classical randomized control groups. Therefore, considering low-risk concurrent controls might also be acceptable under certain circumstances, particularly when clinical equipoise is far from a coin flip [20]. In the current situation, where uncontrolled real-world evidence is gaining widespread acceptance, it may also be worthwhile to use risk-based allocation in clinical trials more extensively.

In Brazil, individuals with comorbidities accounted for the majority of COVID-19 hospitalizations [21], and the burden in this group also increased across successive COVID-19 waves [22]. Studies have shown that vaccination provided substantial protection against severe COVID-19 outcomes, especially after a booster dose [23]. This study utilized a risk-based allocation of vaccination priority to individuals with comorbidities, resulting in two distinct groups: those who received the vaccine (high-risk of severe COVID-19 outcomes if infected) and those who did not (low-risk comparator). This approach enabled meaningful comparisons across key outcomes despite the lack of randomization and practical, ethical, social, and logistical restrictions [24,25]. The data demonstrate that vaccinated individuals with risk factors had similar rates of hospitalization and death as unvaccinated individuals without risk factors, suggesting that the vaccine effectively offset the elevated baseline risk associated with comorbidities. These findings align with broader real-world studies of CoronaVac in Chile and Brazil, which reported substantial reductions in severe COVID-19 outcomes during periods of different variant circulation [7,13,26,27].

At baseline (D0), both groups exhibited comparable rates of seropositivity, indicating a high prevalence of prior exposure to SARS-CoV-2 in the study population, which is consistent with findings from surveys conducted in Manaus [28,29]. Despite this initial immunity, the significant increase in anti-Spike IgG levels following vaccination, particularly by day 28 and day 90, demonstrates that CoronaVac successfully boosted humoral responses, even among individuals who were previously exposed. However, a decline in antibody titers was observed by D180, consistent with reports of waning immunity following inactivated vaccines [30,31]. Protective antibody titers decrease about a year after vaccination, and the vaccine does not perform as effectively as the BNT162b2 in providing a booster response against the SARS-CoV-2 Delta and Omicron variants [30,31]. Randomized controlled trials initially showed that the vaccine had 50% and 84% efficacy against symptomatic COVID-19 and hospitalizations due to COVID-19, respectively, among healthcare workers before the emergence of the Gamma variant [25]. Evidence from a test-negative study in Manaus during Gamma variant transmission was mixed, with some protection observed after at least one CoronaVac dose against symptomatic SARS-CoV-2 infection, but low estimated effectiveness after the two-dose schedule [26].

Severe outcomes (hospitalization and death) were similarly rare among vaccinated high-risk and unvaccinated low-risk individuals across all time points. This finding suggests that CoronaVac may effectively mitigate the elevated baseline risk conferred by comorbidities, particularly obesity, and reinforces prior evidence that the vaccine is more effective in preventing severe COVID-19 outcomes than in preventing infection itself [27]. Indeed, the most likely approach to understanding the value of COVID-19 vaccines is to analyze their effectiveness against progression to severe disease [32]. Notably, most individuals in the early vaccination group were classified as obese, a condition known to be strongly associated with severe COVID-19, ICU admission, and mortality [33,34]. Further studies are needed to clarify how varying degrees of obesity influence serodetection and immune responses following COVID-19 vaccination.

This study has some limitations. The low denominator in the low-risk control group, which limited our power to further analysis, is due to the progressive rollout of vaccines to the general population and losses to follow-up. The non-randomized design introduces the potential for confounding, although baseline characteristics between groups were generally balanced. Furthermore, the analysis employed a frequentist approach rather than a Bayesian statistic, as initially proposed for this type of trial [35]. Prior asymptomatic SARS-CoV-2 infections may have influenced outcomes, despite baseline serological screening. The study was also limited to a single urban population of working-age adults. Additionally, viral sequencing was not performed, although local epidemiology strongly suggests that Gamma was the predominant circulating variant during the study period [36,37].

In conclusion, this risk-based allocation clinical trial with CoronaVac demonstrated an apparent reduction in the risk of hospitalization and death among individuals with comorbidities, aligning their risk with that of lower-risk, unvaccinated individuals. Beyond the clinical findings, this design serves as a model for rapidly generating actionable evidence under real-world constraints. It should be considered not only in future pandemic response plans but also as a valid routine strategy to generate evidence of the clinical benefit of an intervention, whether for regulatory or public health purposes, thereby providing the best opportunity to those at higher risk.

## Supporting information

S1 Table Description of comorbidities included as priorities for vaccination. Translated from the Ministry of Health, Brazil, 2021 [15].
(DOCX)

S1 Appendix Data dictionary. (XLSX)

S2 Appendix Dataset. (CSV)

S3 Appendix Executive summary of protocol. (DOCX)

S4 Appendix Protocol in Original Language. (PDF)

S5 Appendix Consort checklist. (DOCX)
