# Supplementary material for: Effectiveness of CoronaVac in a pioneer risk-based allocation clinical trial during the COVID-19 pandemic
Source: PLoS One. 2026 Jun 22;21(6):e0351566. doi: 10.1371/journal.pone.0351566 (PMC13286151; doi:10.1371/journal.pone.0351566)
Supplement: S1 Table — Translated from the Ministry of Health, Brazil, 2021 [15]. (DOCX) [file pone.0351566.s001.docx]

**S1 Table. Description of comorbidities included as priorities for vaccination**. Translated from the Ministry of Health, Brazil, 2021 [15].

| **Comorbidity group** | **Description** |
| --- | --- |
| Diabetes mellitus | Any individual with diabetes |
| Severe chronic lung diseases | Individuals with severe lung diseases including chronic obstructive pulmonary disease, cystic fibrosis, pulmonary fibrosis, pneumoconiosis, bronchopulmonary dysplasia, and severe asthma (recurrent use of systemic corticosteroids, previous hospitalization due to asthmatic crisis). |
| Resistant Arterial Hypertension (RHT) | When blood pressure (BP) remains above the recommended targets with the use of three or more antihypertensive drugs from different classes, at maximum recommended and tolerated doses, administered frequently, appropriate dosage, and proven adherence or controlled BP using four or more antihypertensive drugs |
| Stage 3 high blood pressure | Systolic BP ≥180mmHg and/or diastolic BP ≥110mmHg regardless of the presence of target organ damage (TOD) or comorbidity |
| Stage 1 and 2 arterial hypertension with LOA and/or comorbidity | Systolic BP between 140 and 179mmHg and/or diastolic BP between 90 and 109mmHg in the presence of TOD and/or comorbidity |
| Heart failure (HF) | HF with reduced, intermediate, or preserved ejection fraction; in stages B, C, or D, regardless of New York Heart Association functional class |
| *Cor pulmonale* and pulmonary hypertension | Chronic *cor pulmonale*, primary or secondary pulmonary hypertension |
| Hypertensive heart disease | Hypertensive heart disease (left ventricular hypertrophy or dilation, atrial and ventricular overload, diastolic and/or systolic dysfunction, other target organ damage) |
| Coronary syndromes | Chronic coronary syndromes (stable angina pectoris, ischemic heart disease, after Acute Myocardial Infarction, others) |
| Valvular heart disease | Valve lesions with hemodynamic or symptomatic repercussions or with myocardial involvement (aortic stenosis or insufficiency; mitral stenosis or insufficiency; pulmonary stenosis or insufficiency; tricuspid stenosis or insufficiency, and others) |
| Cardiomyopathies and pericardiopathies | Cardiomyopathies of any etiology or phenotype; chronic pericarditis; rheumatic heart disease |
| Diseases of the aorta, great vessels, and arteriovenous fistulas | Aneurysms, dissections, hematomas of the aorta and other large vessels |
| Cardiac arrhythmias | Cardiac arrhythmias with clinical importance and/or associated heart disease (atrial fibrillation and flutter, and others) |
| Congenital heart disease in adults | Congenital heart diseases with hemodynamic repercussions, hypoxemia, cardiac insufficiency, arrhythmias, and commitment myocardial. |
| Valve prostheses and implanted cardiac devices | Patients with biological or mechanical valve prostheses, and implanted cardiac devices (pacemakers, cardio-defibrillators, resynchronizers, medium and long-term circulatory assistance) |
| Cerebrovascular disease | Ischemic or hemorrhagic stroke, transient ischemic attack, vascular dementia |
| Sickle cell anemia | Sickle cell anemia |
| Chronic kidney disease | Chronic kidney disease stage 3 or more (glomerular filtration rate<60 mL/min/1.73m^2^) and nephrotic syndrome. |
| immunosuppressed | Individuals with solid organ or bone marrow transplants; people living with HIV and CD4<350 cells/mm^3^; systemic immune-mediated rheumatic diseases in activity and using a dose of prednisone or equivalent >10 mg/day or receiving pulse therapy with corticosteroids and/or cyclophosphamide; other individuals using immunosuppressants or with primary immunodeficiencies. |
| Morbid obesity | Body mass index (BMI) ≥40 |
| Down's syndrome | Trisomy of chromosome 21 |

Additional notes: For diabetes, we considered a glucose measurement of 200 mg/dL or higher, as well as a prescription for glycemic control medication. For hypertension, the measurement of blood pressure at the site was considered 140 mmHg for systolic blood pressure and/or 90 mmHg for diastolic blood pressure, or the prescription of medication to control hypertension, or a medical report. For immunosuppression, self-report of any immunosuppressive disease, people living with HIV, regardless of CD4 count. Obesity was defined as a body mass index (BMI) of 30 kg/m², measured at the site. For other comorbidities, a medical report describing the comorbidity, along with laboratory and imaging tests, was considered.
