# Supplementary material for: Effectiveness of CoronaVac in a pioneer risk-based allocation clinical trial during the COVID-19 pandemic
Source: PLoS One. 2026 Jun 22;21(6):e0351566. doi: 10.1371/journal.pone.0351566 (PMC13286151; doi:10.1371/journal.pone.0351566)
Supplement: S3 Appendix — (DOCX) [file pone.0351566.s004.docx]

**COVACMANAUS Study**

**Version 5.0 — May 23, 2024
Clinical Research Protocol**

*CONFIDENTIAL*

**Clinical Research Protocol**

Phase IV Study to Evaluate the Effectiveness of the Inactivated Adsorbed Vaccine against COVID-19 (CoronaVac), among Education and Public Security Professionals with Risk Factors for Severity, in Manaus (Amazonas)

**Study Acronym**

COVACMANAUS

**Version:** 5.0 — May 23, 2024

CONFIDENTIAL

The confidential information contained in this document is provided to you as an investigator or member of a research study team, or reviewer within the study approval process. By accepting this document, you agree not to disclose the information contained herein to other parties without proper authorization.

Fundação de Medicina Tropical Dr. Heitor Vieira Dourado

Av. Pedro Teixeira, 25

Manaus – AM 69040-000 – Brazil

# 1 EXECUTIVE SUMMARY

## 1.1 PROTOCOL TITLE

Phase IV Study to Evaluate the Effectiveness of the Inactivated Adsorbed Vaccine against COVID-19 (CoronaVac), among Education and Public Security Professionals with Risk Factors for Severity, in Manaus (Amazonas).

## 1.2 REGISTRATION INFORMATION

| **Category** | **Information** |
| --- | --- |
| Primary registry | ClinicalTrials.gov (NCT04789356) |
| Registration date | March 9, 2021 |
| Financial/material/human resource sponsors | Fundação de Amparo à Pesquisa do Estado do Amazonas (FAPEAM); Instituto Butantan; Universidade do Estado do Amazonas (UEA) |
| Sponsor/Proponent | Fundação de Medicina Tropical Dr. Heitor Vieira Dourado |
| Principal Investigator and public/scientific contact | Marcus Vinícius Guimarães de Lacerda, MD, PhD Fundação de Medicina Tropical Dr. Heitor Vieira Dourado Av. Pedro Teixeira, 25 Manaus, AM, Brazil ZIP 69040-000 Email: marcuslacerda.br@gmail.com |
| Co-Principal Investigator | Maria Paula Gomes Mourão, MD, PhD Universidade do Estado do Amazonas — UEA Av. Djalma Batista, 3578 ZIP: 69050-010 Fundação de Medicina Tropical Dr. Heitor Vieira Dourado Av. Pedro Teixeira, 25 Manaus, AM, Brazil ZIP 69040-000 Email: mariapaula.mourao@gmail.com |

| Scientific title | Phase IV Study to Evaluate the Effectiveness of the Inactivated Adsorbed Vaccine against COVID-19 (CoronaVac), among Education and Public Security Professionals with Risk Factors for Severity, in Manaus (Amazonas) |
| --- | --- |
| Study acronym | COVACMANAUS |
| Recruitment countries | Brazil |
| Conditions/health problems | COVID-19 |
| Interventions — Name | CoronaVac Adsorbed COVID-19 Vaccine (inactivated) |
| Interventions — Description | Dose: 600 SU/dose; Two doses, 28 days apart; Intramuscular (deltoid) |

**Inclusion criteria**

• Adults aged 18 to 49 years;

• Willingness to be followed for the study-defined follow-up period via visits, phone calls, or other digital communication means.

**Exclusion criteria**

• Prior vaccination for COVID-19;

• Diagnosis of COVID-19 in the last 28 days (nasal and oropharyngeal swab);

• History of severe allergic reaction or anaphylaxis to components of the study vaccine;

• Reported fever within 72 hours prior to vaccination (inclusion may be postponed until the participant has been fever-free for 72 hours);

• Suspected or confirmed COVID-19 on the day of vaccination (vaccination may be postponed until 72 hours without symptoms or until the diagnosis is ruled out);

• Use of a live attenuated vaccine within 28 days or an inactivated vaccine within 14 days prior to inclusion, or having immunization scheduled for the first 28 days after inclusion;

• Any other condition that, in the opinion of the principal investigator or medical designee, may put at risk the safety or rights of a potential participant or prevent compliance with the protocol;

• Pregnancy or lactation.

## Study Design

| Study type | Quasi-experimental study |
| --- | --- |
| Allocation type | Quasi-experimental, with two groups: high-risk participants (with comorbidities as a risk factor for severe COVID-19 per the National Plan for Operationalization of Vaccination against COVID-19), vaccination anticipated within the research project vs. low-risk participants (without comorbidities as a risk factor for severe COVID-19 per the National Plan), who will not receive the vaccine within the scope of the research project. |
| Recruitment status | Completed |
| Estimated date of 1st recruitment | March 15, 2021 |
| Target sample size | 10,156 participants, allocation ratio 1:1 between groups |

**Primary outcomes**

• Effectiveness: Incidence density of moderate and severe clinical COVID-19 cases (severity ≥4 per WHO clinical progression scale) starting in the second week after the second vaccine dose.

**Secondary outcomes**

**Effectiveness**

• Incidence density of moderate and severe clinical COVID-19 cases (severity ≥4 per WHO scale) from the first dose;

• Incidence density of moderate and severe clinical COVID-19 cases (severity ≥4 per WHO scale) from the second dose;

• Incidence density of severe clinical COVID-19 cases (severity ≥6 per WHO scale) from the second dose;

• Median clinical progression scores among moderate and severe cases;

• Incidence density of virologically confirmed clinical COVID-19 cases;

• Incidence density of deaths confirmed as COVID-19;

• Incidence density of hospitalization for any cause;

• All-cause mortality density.

**Safety**

• Frequency of adverse events requiring medical care up to 7 days after each vaccine dose (D7 and D35).

**Immunogenicity**

• Immune response to vaccination in a subgroup of participants before each vaccination and every 3 months up to 12 months of follow-up;

• Cell-mediated immune response to vaccination in a subgroup of participants before each vaccination and every 3 months up to 12 months of follow-up;

• Presence of antibodies against SARS-CoV-2 before each vaccination and every 3 months up to 12 months of follow-up.

**Erratum / Amendment to the COVACMANAUS Protocol**

**Version 5.0 (23 May 2024)**

**Section:** Sample size calculation

**Correction:**
In the sample size calculation, the significance level was incorrectly reported as *“alfa unicaudal de 0,5%”*. The correct value used in the calculation was a **one-sided alpha of 5% (α = 0.05)**.

This represents a **notation error** in the protocol text. The sample size estimation (total of 10,156 participants, 1:1 allocation, 90% power, assumed incidences of 0.41% and 1.03%, and 30% annual loss rate) was conducted using **α = 0.05**, consistent with standard practice and with the analyses reported in the manuscript.

This correction does **not** affect the study design, ethical approval, conduct of the study, or interpretation of the results.
