# Supplementary material for: Effectiveness of CoronaVac in a pioneer risk-based allocation clinical trial during the COVID-19 pandemic
Source: PLoS One. 2026 Jun 22;21(6):e0351566. doi: 10.1371/journal.pone.0351566 (PMC13286151; doi:10.1371/journal.pone.0351566)
Supplement: S4 Appendix — (PDF) [file pone.0351566.s005.pdf]

Protocolo de pesquisa clínica	Estudo de Fase IV para Avaliação de Efetividade da Vacina Adsorvida Inativada contra COVID-19 Coronavac, entre Profissionais da Educação e de Segurança Pública com Fatores de Risco para Gravidade, em Manaus (Amazonas)
Acrônimo do Estudo	COVACMANAUS
Versão:	5.0 23 de maio de 2024

CONFIDENCIAL

As informações confidenciais contidas neste documento são fornecidas para você como um investigador ou membro de uma equipe de pesquisa do estudo ou revisor dentro do processo de aprovação do estudo. A sua aceitação deste documento se caracteriza em um acordo no qual você se compromete a não revelar as informações aqui contidas para outras partes sem a devida autorização.

Fundação de Medicina Tropical Dr. Heitor Vieira Dourado
Av. Pedro Teixeira, 25
Manaus – AM 69040-000 – Brasil

CONTEÚDO

1 Resumo Executivo 6
1.1 Título do protocolo 6
1.2 Informação de registro 6
1.3 Histórico de versões 10
1.4 Financiamento 11
1.5 Responsáveis pelo protocolo 11
1.5.1 Contribuições para o protocolo 11
1.5.2 Papel do Investigador e financiadores 11
1.5.3 Comitês do estudo 12
2 Introdução 12
2.1 Estado atual do conhecimento sobre o tema do projeto 12
2.1.1 Infecções por coronavírus em seres humanos 12
2.1.2 A emergência de SARS-CoV-2 13
2.1.3 Apresentação clínica 13
2.1.4 Epidemiologia da COVID-19 15
2.1.5 Desenvolvimento de vacinas contra a COVID-19 18
2.2 Objetivos 23
2.2.1 Objetivo primário 23
2.2.2 Objetivos secundários 23
2.2.2.1 Avaliação da efetividade 23
2.2.2.2 Avaliação de Segurança 24
2.2.2.3 Avaliação da Imunogenicidade 24
2.2.3 Objetivos exploratórios 24
2.3 Desenho geral do estudo 25
3 Métodos 26

3.1 Centro de estudo 26

3.2 Participantes 26

3.2.1 Critérios de inclusão 26

3.2.2 Critérios de exclusão 26

3.3 Desfechos 27

3.3.1 Desfechos primários 27

3.3.2 Desfechos secundários 27

3.3.3 Desfechos exploratórios 28

3.4 Procedimentos 29

3.4.1 Vacina adsorvida Coronavac (vírus inativado) 29

3.4.1.1.1 Dose/Administração/Armazenamento 29

3.4.1.1.2 Lotes da vacina 29

3.4.1.1.3 Indicações de uso 29

3.5 Procedimentos para o participante 30

3.5.1 Acompanhamento dos participantes 30

3.5.2 Descrição dos procedimentos do estudo por visita 30

3.5.2.1 Procedimentos do estudo 30

3.5.2.1.1 Triagem 31

3.5.2.1.2 Visita de vacinação (Vac 1 e Vac2) 31

3.5.2.1.3 Contato telefônico (Ct)....32

3.5.2.1.4 Visitas de retorno (Vr)....33

3.5.2.1.4.1 Procedimentos: 33

3.5.2.1.5 Visita de Hospitalização (Vh)....33

3.5.2.1.6 Visita não-programada (VNP) 33

3.5.2.1.7 Captura de Eventos Adversos 35

3.5.3 Procedimento em caso de perda de visita ou contato 36

3.6 Tabela de procedimentos do estudo 37
3.6.1 Teste de Neutralização Viral (VNT) 37
3.6.2 Imunofenotipagem celular para Populações de Memória 37
3.6.3 Teste de antígeno 38
3.6.3.1 Coleta do swab nasal 39
3.6.4 RT-PCR diagnóstico de Covid-19 39
3.6.4.1 Coleta dos swabs nasal e orofaríngeo 39
3.6.4.2 Detecção de SARS-CoV-2 P.1 / VOCs por RT – PCR 40
3.6.4.3 Amplificação e sequenciamento de SARS-CoV-2 40
3.6.5 Testes laboratoriais complementares 41
3.7 Critérios e procedimentos para exclusão do participante 44
3.8 Tamanho amostral 44
3.8.1 Cálculo de tamanho amostral e poder estatístico 44
3.9 População fonte do estudo e área de recrutamento 46
3.10 Coleta e gerenciamento de dados 46
3.11 programa de análise e processamento de dados 46
3.12 Plano de Análise 47
3.12.1 Efetividade 47
3.12.2 Segurança 48
3.12.3 Imunogenicidade 48
3.12.4 Gerenciamento de dados faltantes/ausentes 49
3.13 Monitoramento de dados do estudo 49
3.13.1 Comitê de monitoramento de dados e segurança 49
3.13.2 Análises e revisão interina 50
3.13.3 Definições de caso de COVID-19 51

3.14 Garantia/controle de qualidade de dados 51
3.14.1 Monitoramento clínico 51
3.14.2 Acesso aos documentos fonte 51
4 Ética e publicações 52
4.1 Aprovações para a realização da pesquisa 52
4.1.1 Declaração do marco regulatório do estudo 52
4.1.2 Aprovações do estudo 52
4.2 Emendas ao protocolo 52
4.3 Consentimento livre e esclarecido 52
4.3.1 Implementação e documentação do consentimento livre e esclarecido 52
4.3.2 amostras biológicas 54
4.3.2.1 Local de realização dos testes previstos no protocolo 54
4.3.2.2 Armazenamento de amostras biológicas e uso de biorrepositório 54
4.4 Descrição dos riscos 55
4.4.1 Detalhamento dos riscos do estudo 55
4.4.1.1 Punção venosa 55
4.4.1.2 *Swab* nasal 55
4.4.2 Descrição dos benefícios antecipados ao participante do estudo 56
4.4.3 Descrição da relação entre os riscos potenciais e benefícios antecipados 56
4.4.4 Descrição de custos e justificativa de reembolsos ou compensações 56
4.5 Confidencialidade 56
4.6 Acesso aos dados 57
4.6.1 Divulgação dos resultados ao público 57
4.6.2 Comunicado aos participantes dos achados do estudo 58
ANEXO A 59
ANEXO B 60

1 Resumo Executivo

1.1 Título do protocolo

Estudo de Fase IV para Avaliação de Efetividade da Vacina Adsorvida Inativada contra COVID-19 Coronavac, entre Profissionais da Educação e de Segurança Pública com Fatores de Risco para Gravidade, em Manaus (Amazonas)

1.2 Informação de registro

Categoria	Informação
Registro Primário	ClinicalTrials.gov (NTC04789356)
Data de registro	09 de março de 2021
Fontes de apoio financeiro, material ou com recursos humanos	Fundação de Amparo à Pesquisa do Estado do Amazonas (FAPEAM)<br>Instituto Butantan<br>Universidade do Estado do Amazonas (UEA)
Proponente	Fundação de Medicina Tropical Dr. Heitor Vieira Dourado
Investigador principal e contato para questões públicas e científicas	Marcus Vinícius Guimarães de Lacerda, MD, PhD<br>Fundação de Medicina Tropical Dr. Heitor Vieira Dourado<br>Av. Pedro Teixeira, 25<br>Manaus, AM, Brasil CEP 69.040-000<br>E-mail: marcuslacerda.br@gmail.com
Co-investigadora principal	Maria Paula Gomes Mourão, MD, PhD<br>Universidade do Estado do Amazonas – UEA<br>Av. Djalma Batista, 3578 CEP: 69050-010;<br><br>Fundação de Medicina Tropical Dr. Heitor Vieira Dourado<br>Av. Pedro Teixeira, 25<br>Manaus, AM, Brasil CEP 69.040-000<br>E-mail: mariapaula.mourao@gmail.com

Título científico		Estudo de Fase IV para Avaliação de Efetividade da Vacina Adsorvida Inativada contra COVID-19 Coronavac, entre Profissionais da Educação e de Segurança Pública com Fatores de Risco para Gravidade, em Manaus (Amazonas)
Acrônimo do estudo		COVACMANAUS
Países de recrutamento		Brasil
Condições ou problemas de saúde		COVID-19
Intervenções	Nome	Vacina (CoronaVac) Adsorvida COVID-19 (inativada)
	Descrição	Dose 600 SU/dose; Duas doses, sendo separadas por 28 dias<br>Via: Intramuscular (deltóide)
Critérios de inclusão		Adultos entre 18 anos e 49 anos de idade;<br>Demonstrar disponibilidade de ser acompanhado durante o tempo de seguimento definido no estudo, por meio de visitas, contatos telefônicos ou outros meios de comunicação digital.
Critérios de exclusão		Vacinação prévia para COVID-19;<br>Diagnóstico de COVID-19 nos últimos 28 dias (*swab* nasal e orofaríngeo);<br>História de reação alérgica grave ou anafilaxia aos componentes da vacina de estudo;<br>Relato de febre nas 72 horas que antecedem à vacinação (a inclusão pode ser adiada até que o participante complete 72 horas sem febre);<br>Caso possível ou confirmado de COVID-19 no dia da vacinação (nesta situação, a vacinação pode ser adiada até que o participante complete 72 horas sem sintomas ou seja descartado o diagnóstico);<br>Ter recebido vacina com vírus vivo atenuado nos últimos 28 dias ou vacina inativada nos últimos 14 dias que antecedem a sua inclusão no estudo, ou ainda ter imunização programada para os primeiros 28 dias após sua inclusão no estudo;

	Qualquer outra condição que, na opinião do investigador principal ou do seu representante médico, possa colocar em risco a segurança ou os direitos de um participante em potencial ou que o impeça de cumprir com este protocolo;<br>Gravidez ou lactante.
Tipo de estudo	Estudo Quase-Experimental
Tipo de alocação	Estudo Quase-Experimental, com dois grupos:<br>Participantes com **alto risco** (com presença de comorbidades como fator de risco para COVID-19 grave de acordo com o Plano Nacional de Operacionalização da Vacinação Contra a COVID-19), **com antecipação** da vacina no âmbito do projeto de pesquisa *versus* participantes com **baixo risco** (sem comorbidades como fator de risco para COVID-19 grave de acordo com o Plano Nacional de Operacionalização da Vacinação Contra a COVID-19), que **não** receberão a vacina no âmbito do projeto de pesquisa.
Status de recrutamento	Finalizado.
Data provável do 1º recrutamento	15 de março de 2021
Tamanho da amostra alvo	10.156 participantes, com razão de alocação de 1:1 entre grupos
Desfechos primários	O desfecho primário de efetividade é a densidade de incidência de casos clínicos moderados e graves de COVID-19 (intensidade 4 ou superior conforme a escala de progressão clínica da OMS), depois da segunda semana após a segunda dose da vacina.

Desfechos secundários	Efetividade:<br>Densidade de incidência de casos clínicos moderados e graves de COVID-19 (intensidade 4 ou superior conforme a escala de progressão clínica da OMS) a partir da **primeira dose**<br>Densidade de incidência de casos clínicos moderados e graves de COVID-19 (intensidade 4 ou superior conforme a escala de progressão clínica da OMS) a partir da **segunda dose**<br>Densidade de incidência de casos clínicos **graves** de COVID-19 (intensidade 6 ou superior conforme a escala de progressão clínica da OMS) a partir da segunda dose<br>Mediana dos escores de progressão clínica entre os casos moderados e graves<br>Densidade de incidência de casos clínicos confirmados virologicamente como COVID-19<br>Densidade de incidência de óbitos confirmados como COVID-19<br>Densidade de incidência de internação hospitalar por qualquer causa<br>Densidade de mortalidade por qualquer causa<br>Segurança:<br>Frequência de eventos adversos associados à necessidade de atendimento médico até 7 dias após cada dose da vacina (D7 e D35).<br>Imunogenicidade:<br>Resposta imune à vacinação em um subgrupo de participantes antes de cada vacinação e a cada 3 meses, até 12 meses de seguimento.<br>Resposta imune à vacinação mediada por células em um subgrupo de participantes antes de cada vacinação e a cada 3 meses, até 12 meses de seguimento.<br>Presença de anticorpos contra SARS-CoV-2 antes de cada vacinação e a cada 3 meses, até 12 meses de seguimento.

1.3 Histórico de versões

Versão	Data	Comentário
1.0	26-fev-2021	Versão inicial
2.0	09-mar-2021	Alterações:<br>Adição de trecho declarando que o grupo de participantes que não recebeu a vacina no âmbito do estudo receberá, após os 12 meses, e se não tiver recebido qualquer outra vacina disponível, receberá a vacina do estudo, exatamente como oferecida no grupo com vacina (duas doses de CoronaVac com intervalo de 28 dias – podendo ser 7 dias antes ou depois).<br>Adição do registro do estudo no ClinicalTrials.gov
3.0	30-mar-2021	Ampliação da população do estudo para profissionais de educação da rede privada, incluindo ensino fundamental.<br>Inserção de co-investigadora principal e instituição colaboradora.<br>Ajustes na tabela de procedimentos do estudo (D35 – a janela desta visita foi ampliada para +/- 7 dias em virtude da possibilidade de antecipação da segunda dose também em 7 dias).
4.0	19-ago-2021	Inserção de 4 co-investigadores na pesquisa (Alex Martins, Fabíola Mendonça da Silva Chui, Maurício Lacerda Nogueira e Guilherme Campos) conforme ANEXO B;<br>Alteração da janela de visitas programadas presenciais D180, D270 e D360 para ± 14 dias. As demais visitas programadas não-presenciais manterão a mesma janela de ± 7 dias, conforme Tabela 1.<br>Descrição da metodologia de testes de laboratório para *swab* nasal (RT-PCR e teste de antígeno), soro (anticorpos) e PBMC;<br>Inserção da descrição de visita não-programada e de exclusão de participantes;
5.0	23-mai-2024	Inserção do ISGlobal nas contribuições para o protocolo (item 1.5.1);<br>Adicionado o item 3.6.5. Teste laboratoriais complementares para medir o reconhecimento de variantes pelos anticorpos induzidos pela vacina,

	avaliar o escape de neutralização de anticorpos de VoC, e caracterizar a duração das respostas imunes e correlatos de proteção;<br>Inserção de informações no intem 4.3.2.2. referentes aos testes complementares que serão realizados com as amostras biológicas armazenadas em biorrepositorio, as quais serão enviadas ao ISGlobal, em Barcelona, Espanha. As amostras que serão enviadas não contêm nenhum material genético viável dos participantes.

1.4 Financiamento

Este estudo tem financiamento da Fundação de Amparo à Pesquisa do Estado do Amazonas (FAPEAM).

1.5 Responsáveis pelo protocolo

Os detalhes dos responsáveis pelo protocolo estão detalhados no Anexo B.

1.5.1 Contribuições para o protocolo

Este protocolo de pesquisa foi escrito pelas equipes da Fundação de Medicina Tropical Dr. Heitor Vieira Dourado, do Instituto Leônidas & Maria Deane (Fiocruz Amazônia), do Instituto Butantan e da Universidade do Estado do Amazonas (UEA). O protocolo recebeu contribuições do Institute for Global Health (ISGlobal).

1.5.2 Papel do Investigador e financiadores

O financiador não terá influência no desenho e execução, na análise, na interpretação de dados e na publicação dos resultados do estudo. A Fundação de Medicina Tropical Dr. Heitor Vieira Dourado, ligada à Secretária de Estado da Saúde do Amazonas (SES-AM), assume as responsabilidades de proponente do estudo em conformidade com as Boas Práticas Clínicas. Trata-se de um estudo de iniciativa do investigador. Desta forma, o investigador fará a administração dos recursos financeiros para garantir o desempenho adequado do estudo e da plataforma para entrada de dados. O

investigador também obterá as aprovações necessárias junto ao sistema CEP/CONEP. O Instituto Butantan fornecerá o produto sob investigação, após a devida notificação do estudo à ANVISA. As análises de dados serão realizadas por uma equipe da Fundação de Medicina Tropical Dr. Heitor Vieira Dourado e do Instituto Butantan, que terão acesso às bases de dados originais e poderão solicitar ou realizar análises adicionais conforme este protocolo, complementado por um plano de análise estatístico a ser finalizado antes do congelamento da base de dados. A publicação dos resultados do estudo será feita de comum acordo entre investigador e demais colaboradores, sem prejuízo do direito do investigador de apresentar o estudo e suas conclusões em revistas e encontros científicos.

1.5.3 Comitês do estudo

Um Comitê de Monitoramento de Dados e Segurança (CMDS) será constituído para o estudo.

2 Introdução

2.1 Estado atual do conhecimento sobre o tema do projeto

2.1.1 Infecções por coronavírus em seres humanos

Os coronavírus são vírus RNA de genoma relativamente grande ≈30kB que se apresentam de forma ubíqua na natureza em diversas espécies animais, inclusive causando doenças em animais domésticos e de criação. Antes do surgimento da COVID-19, foram identificados seis coronavírus capazes de causar infecção em seres humanos. Na década de 1960, dois coronavírus causadores de quadros de resfriado comum foram identificados, HCoV-OC43 e HCoV-229E. No presente século, foram acrescentados mais dois coronavírus como agentes associados a resfriado comum, HCoV-NL63 e HCoV-HKU1. Todos eles, exceto o último, têm proximidade genética com coronavírus de morcegos, que podem ter sido sua origem. Os dois coronavírus restantes, também identificados no presente século, são o SARS-CoV-1, associado a surtos de síndrome respiratória aguda grave (SRAG) que

começaram em 2002, e o MERS-CoV encontrado em 2012, associado também a quadros de SRAG em pessoas em contato com camelos e trabalhadores de saúde de locais que atendiam esses pacientes[1].

2.1.2 A emergência de SARS-CoV-2

No final do mês de dezembro de 2019, um agregado de pacientes com pneumonia foi identificado em associação com um mercado na cidade de Wuhan, na Província de Hubei, na China. Em amostras coletadas de lavagem bronco-alveolar de três desses pacientes foi identificado um coronavírus diferente daqueles conhecidos como infecciosos para os seres humanos. O novo coronavírus foi identificado como um betacoronavírus, assim como SARS-CoV-1, com o qual possui maior proximidade genética[2]. Esse vírus foi denominado posteriormente SARS-CoV-2

SARS-CoV-2 é um vírus esférico composto de um RNA de fita simples associado a uma nucleoproteína dentro de uma cápside proteica recoberta por um envelope com glicoproteínas projetadas ao exterior em forma de espícula, ou Spike em inglês. A proteína Spike é fundamental para sua replicação, de forma análoga ao SARS-CoV-1, e os anticorpos neutralizantes estão direcionados principalmente contra ela[3].

A transmissão da infecção de SARS-CoV-2 é principalmente por gotículas e fômites, como muitos outros vírus respiratórios. O vírus pode se manter viável por várias horas ou dias, em diferentes superfícies[4]. A replicação em vias aéreas superiores facilita a transmissão pessoa a pessoa e a contaminação de ambientes fechados.

2.1.3 Apresentação clínica

---

[1] de Wilde AH et al. Host Factors in Coronavirus Replication. In Roles of Host Gene and Non-coding RNA Expression in Virus Infection 2017. p. 1–42.

[2] Zhu Net al. A Novel Coro navirus from Patients with Pneumonia in China, 2019. N Engl J Med. 2020 Feb 20;382(8):727–33.

[3] Mousavizadeh L et al. Genotype and phenotype of COVID-19: Their roles in pathogenesis. J Microbiol Immunol Infect [Internet]. 2020 Ma.

[4] van Doremalen N, Bushmaker T, Morris DH, Holbrook MG, Gamble A, Williamson BN, et al. Aerosol and Surface Stability of SARS-CoV-2 as Compared with SARS-CoV-1. N Engl J Med [Internet]. 2020 Apr 16;382(16):1564–7.

Desde os relatos iniciais de casos da doença na cidade de Wuhan, o espectro clínico de COVID-19 foi bem caracterizado. A apresentação é de um quadro febril com sintomas respiratórios associados, como tosse, que pode evoluir para pneumonia bilateral com comprometimento difuso, que pode progredir para síndrome de angústia respiratória aguda, lesão cardíaca e falência múltipla de órgãos. A evolução para gravidade é mais frequente em pacientes de idade avançada e aqueles com comorbidades, especialmente hipertensão, diabetes e doença cardiovascular[5]. A associação entre gravidade e condições médicas subjacentes foi confirmada em outras séries de casos[6].

Os casos de COVID-19 se apresentam predominantemente com febre, que pode estar acompanhada de fadiga, tosse não produtiva ou com expectoração. Outros sintomas incluem mialgia, anorexia, sensação de aperto torácico e dispneia. Sintomas menos frequentes são náusea e vômito, diarreia, cefaleia, dor na faringe, calafrios e dor abdominal[7]. Cabe destacar entre os sintomas leves e moderados as alterações de olfato e paladar, que se apresentaram mais comumente entre mulheres[8]. Em crianças, os quadros de COVID-19 geralmente são mais leves e menos frequentes[9,10].

---

[5] Huang C et al. Clinical features of patients infected with 2019 novel coronavirus in Wuhan, China. Lancet [Internet]. 2020 Feb;395(10223):497–506.

[6] Chow N et al. Preliminary Estimates of the Prevalence of Selected Underlying Health Conditions Among Patients with Coronavirus Disease 2019 — United States, February 12–March 28, 2020. MMWR Morb Mortal Wkly Rep [Internet]. 2020 Apr 3;69(13):382–6.

[7] Zhu J et al. Clinical characteristics of 3,062 COVID-19 patients: a meta-analysis. J Med Virol [Internet]. 2020 Apr 15; 4

[8] Lechien JR et al. Olfactory and gustatory dysfunctions as a clinical presentation of mild-to-moderate forms of the coronavirus disease (COVID-19): a multicenter European study. Eur Arch Oto-Rhino-Laryngology [Internet]. 2020 Apr 6;

[9] Bialek S et al. Coronavirus Disease 2019 in Children — United States, February 12–April 2, 2020. MMWR Morb Mortal Wkly Rep [Internet]. 2020 Apr 10;69(14):422–6.

[10] Dong Y et al. Epidemiology of COVID-19 Among Children in China. Pediatrics [Internet]. 2020 Mar 16;e20200702.

A identificação rotineira de casos assintomáticos não é realizada atualmente, ainda que existam relatos de casos índice nessa condição, demonstrando a ocorrência de transmissão eficiente[11].

2.1.4 EPIDEMIOLOGIA DA COVID-19

Em fevereiro de 2021, o número de casos confirmados de COVID-19 no mundo ultrapassou os 112 milhões de pessoas diagnosticadas e com mais de dois milhões de óbitos. Esses dados são atualizados diariamente pela Organização Mundial de Saúde (https://covid19.who.int/) que permite ainda avaliar a expansão geográfica da pandemia, que atualmente atingiu quase a totalidade de países e territórios no mundo. A região das Américas é a mais atingida, com quase metade dos casos e dos óbitos relatados. Os Estados Unidos e o Brasil são os países mais atingidos na região e no mundo, em números absolutos.

Muitos países europeus e asiáticos apresentaram ressurgimento de casos COVID-19, consistente com a permanência de uma grande proporção da indivíduos suscetíveis à infecção, após a primeira onda epidêmica[12]. Na Europa, a Itália, França, Reino Unido, Alemanha e Espanha experimentaram um surto inicial por volta de março de 2020, seguido por queda considerável dos casos em maio de 2020 e novo pico em novembro de 2020 (https://coronavirus.jhu.edu/data/new-cases). No Brasil a situação foi semelhante, e a cidade de Manaus tem sofrido desde dezembro de 2020 um aumento significante de casos, com sobrecarga do sistema de saúde.

Os novos picos são acompanhados por novo aumento proporcional no número de óbitos[13]. Além disto, sabe-se que embora eficazes, os títulos dos anticorpos produzidos naturalmente podem decair

---

[11] Huang L et al. Rapid asymptomatic transmission of COVID-19 during the incubation period demonstrating strong infectivity in a cluster of youngsters aged 16-23 years outside Wuhan and characteristics of young patients with COVID-19: A prospective contact-tracing study. J Infect [Internet]. 2020 Jun;80(6):e1–13.

[12] Diaz RS, Vergara TRC. The COVID-19 second wave: A perspective to be explored. Braz J Infect Dis. 2020 Dec 26:101537.

[13] Long QX. et al. Clinical and immunological assessment of asymptomatic SARS-CoV-2 infections. Nat Med. 2020 doi: 10.1038/s41591-020-0965-6. Aug;26(8):1200-1204.

rapidamente, permitindo que os indivíduos sejam reinfectados em um curto período de tempo[14,15,16,17]. Assim, é concebível que a segunda onda pode representar muitos casos de reinfecção e populações mais jovens expostas tendem a ser mais sintomáticas e/ou graves, devido à redução da imunidade específica, ou pela deriva antigênica devida à evolução viral[18]. É possível, ainda, que alguns indivíduos infectados não desenvolvam imunidade protetora ou precisem de múltiplas infecções para desenvolver este tipo de proteção[19].

Desde a primeira caracterização molecular de SARS-CoV-2, o vírus evoluiu constantemente por meio de mutações no seu genoma, identificadas em pesquisas recentes[20,21,22.] Múltiplas variantes do vírus circulam globalmente. Contudo, três variantes (B.1.1.7, B.1.351 e P.1) têm preocupado os especialistas, pois parecem se disseminar mais rapidamente, o que pode levar a novas ondas de transmissão. A variante B.1.1.7 foi identificada pela primeira vez no Reino Unido e foi associada com maior transmissibilidade[23]. Em janeiro de 2021, as evidências apontaram que a variante B.1.1.7 pode

---

[14] To KK et al. COVID-19 re-infection by a phylogenetically distinct SARS-coronavirus-2 strain confirmed by whole genome sequencing. Clin Infect Dis. 2020 doi: 10.1093/cid/ciaa1275. Aug 25:ciaa1275.

[15] Larson D et al. Case of Early Re-infection with SARS-CoV-2 [published online ahead of print, 2020 Sep 19] Clin Infect Dis. 2020 doi: 10.1093/cid/ciaa1436. ciaa1436

[16] Tillett RL et al. Genomic evidence for reinfection with SARS-CoV-2: a case study. Lancet Infect Dis. 2020 doi: 10.1016/S1473-3099(20)30764-7. Oct 12.

[17] Wen J., Cheng Y., Ling R., Dai Y., Huang B., Huang W. Antibody-dependent enhancement of coronavirus. Int J Infect Dis. 2020;100:483–489. doi: 10.1016/j.ijid.2020.09.015. Nov, Epub 2020 Sep 11. PMID: 32920233; PMCID: PMC7483033

[18] Bonifácio LP et al . Are SARS-CoV-2 reinfection and Covid-19 recurrence possible? a case report from Brazil. Rev. Soc. Bras. Med. Trop. [Internet]. 2020 [cited 2021 Feb 22] ; 53: e20200619..

[19] Diaz RS, Vergara TRC. The COVID-19 second wave: A perspective to be explored. Braz J Infect Dis. 2020 Dec 26:101537.

[20] Cheng L et al Functional alterations caused by mutations reflect evolutionary trends of SARS-CoV-2. Brief Bioinform. 2021 Feb 13:bbab042.

[21] Tang X et al. On the origin and continuing evolution of SARS-CoV-2. Natl Sci Rev. 2020 Mar 3:nwaa036.

[22] Grubaugh ND et al. Public health actions to control new SARS-CoV-2 variants. Cell. 2021 Jan 29:S0092-8674(21)00087-8.

[23] Galloway SE et al. Emergence of SARS-CoV-2 B.1.1.7 Lineage — United States, December 29, 2020–January 12, 2021. MMWR Morb Mortal Wkly Rep 2021;70:95–99

estar associada a uma maior letalidade[24,25,26,27]. Esta variante já pode ser encontrada em outros países[28,29]. A variante B.1.351 (ou 501Y.V2) surgiu de forma independente da B.1.1.7, mas compartilha algumas mutações. Foi identificada pela primeira vez na Zâmbia e na África do Sul, em dezembro de 2020, sendo aparentemente a variante predominante neste país. Atualmente, essa variante pode ser encontrar em outros países[30]. Não há evidências que sugiram que esta variante tenha qualquer impacto na gravidade da doença. A variante P.1 surgiu no Brasil e foi identificada pela primeira vez em quatro viajantes do Brasil, testados durante triagem de rotina no aeroporto de Haneda, Japão[31]. Um estudo relatou um grupo de casos em Manaus em que a variante P.1 foi identificada em 42% das amostras sequenciadas no final de dezembro de 2020[32,33,34]. Atualmente, mais de 90% dos casos em Manaus foram associados à P.1 (FG Naveca, comunicação pessoal). Naveca e colaboradores[22] documentaram, recentemente, dois casos em que os indivíduos foram

[24] Kidd M et al. S-variant SARS-CoV-2 lineage B1.1.7 is associated with significantly higher viral loads in samples tested by ThermoFisher TaqPath RT-qPCR. J Infect Dis. 2021 Feb 13:jiab082.

[25] Brookman S et al. Effect of the new SARS-CoV-2 variant B.1.1.7 on children and young people. Lancet Child Adolesc Health. 2021 Feb 10:S2352-4642(21)00030-4.

[26] Graham C et al. Impact of the B.1.1.7 variant on neutralizing monoclonal antibodies recognizing diverse epitopes on SARS-CoV-2 Spike. bioRxiv [Preprint]. 2021 Feb 3:2021.02.03.429355.

[27] Villoutreix BOet al. In Silico Investigation of the New UK (B.1.1.7) and South African (501Y.V2) SARS-CoV-2 Variants with a Focus at the ACE2-Spike RBD Interface. Int J Mol Sci. 2021 Feb 8;22(4):1695.

[28] Umair M et al. Importation of SARS-CoV-2 Variant B.1.1.7 in Pakistan. J Med Virol. 2021 Feb 11.

[29] Washington NL et al. Genomic epidemiology identifies emergence and rapid transmission of SARS-CoV-2 B.1.1.7 in the United States. medRxiv [Preprint]. 2021 Feb 7:2021.02.06.21251159.

[30] Tang JW et al. Introduction of the South African SARS-CoV-2 variant 501Y.V2 into the UK. Journal of infection. January 17, 2021.

[31] Fujino T et al.Novel SARS-CoV-2 Variant Identified in Travelers from Brazil to Japan. Emerg Infect Dis. 2021 Feb 10;27(4). doi: 10.3201/eid2704.210138.

[32] Faria NR et al.Genomic characterisation of an emergent SARS-CoV-2 lineage in Manaus: preliminary findings. Virological.

[33] Buss LF et al. Three-quarters attack rate of SARS-CoV-2 in the Brazilian Amazon during a largely unmitigated epidemic.Science. 2020; 371: 288-292

[34] Fontanet A et al. COVID-19 herd immunity: where are we?. Nat Rev Immunol. 2020; 20: 583-584

reinfectados com a nova variante. Em um indivíduo a reinfecção foi causada por P.1 e, no outro, por P.2, outra variante emergente.

O comportamento humano é um fator decisivo para o desenvolvimento de novas ondas de transmissão. Os governos estaduais e locais, bem como os próprios indivíduos, diferem quanto ao seu comportamento quanto às medidas preventivas. Alguns seguem as precauções contra a transmissão de COVID-19, como distanciamento físico, lavagem das mãos e uso de máscara[35]. Porém, outros não são rigorosos na adoção dessas medidas e não evitam atividades de alto risco. Locais onde as pessoas vivem ou trabalham juntas (famílias multigeracionais, instituições de longa permanência, prisões e alguns tipos de negócios) tendem a propiciar uma maior disseminação do vírus[36]. Surtos de coronavírus em lares de idosos e grandes aglomerações, nas quais uma ou mais pessoas infectadas transmitem o vírus a muitas outras, continuam a ocorrer. Profissionais da saúde, educadores e agentes de segurança são populações que têm sofrido o impacto da alta transmissibilidade do vírus e suas variantes[37]. Locais com transmissão elevada do vírus, e concomitante alta exposição da população, são os locais com maior predisposição ao surgimento de novas variantes. A exemplo disso estão Manaus, Londres e África do Sul onde foram identificadas as novas variantes já descritas. Logo, a probabilidade do surgimento de novas variantes do vírus SARS-Cov-2 continua sendo em locais com estas características, especialmente na cidade de Manaus. Assim, o constante monitoramento do surgimento de novas variantes que venham a surgir fruto de escape do vírus à resposta imunológica, produzida pela vacinação ou pela infecção natural, é mandatório.

2.1.5 Desenvolvimento de vacinas contra a COVID-19

[35] Howard J. An evidence review of face masks against COVID-19. Proceedings of the National Academy of Sciences Jan 2021, 118 (4) e2014564118.

[36] Interim Guidance on Management of Coronavirus Disease 2019 (COVID-19) in Correctional and Detention Facilities. https://www.cdc.gov/coronavirus/2019-ncov/community/correction-detention/guidance-correctional-detention.html

[37] CDC. Emerging SARS-CoV-2 Variants. https://www.cdc.gov/coronavirus/2019-ncov/more/science-and-research/scientific-brief-emerging-variants.html

Desde a emergência de COVID-19, diversos esforços foram realizados para desenvolver uma vacina, com velocidade nunca antes vista. Diferentes tecnologias têm sido utilizadas, sendo que em menos de 6 meses, diversos produtos vacinais já tinham chegado à fase clínica. Alguns deles, como é o caso da vacina desenvolvida pela farmacêutica chinesa Sinovac Biotech, beneficiaram-se da experiência anterior no desenvolvimento de vacina contra o SARS-CoV-1, que teve sua fase I realizada em 2004[38].

Outros realizaram adaptações de desenvolvimentos contra MERS-CoV, como é o caso da vacina usando o vetor ChAdOx1, que teve sua fase I executada em 2018[39]. Em geral, as tecnologias mais usadas são vacinas com vírus inativados, as de vetores virais (adenovírus, sarampo e outros), que podem ser replicantes ou não, de ácidos nucléicos (DNA e RNA), proteicas e de subunidade por recombinação ou síntese, e partículas semelhantes a vírus (VLP). Boa parte das vacinas fundamentam-se unicamente na proteína Spike ou epítopos dessa proteína, em particular a RBD[40] . Um número muito limitado de candidatos vacinais foca outras proteínas virais, o que representa um potencial risco para aquelas que só geram resposta contra a proteína Spike ou suas regiões, em caso de mutações que tornem a resposta protetora insuficiente.

Cinco vacinas são atualmente aprovadas para uso na prevenção da infecção por SARS-CoV-2: Pfizer-BioNTech, Moderna, CoronaVac, Oxford-AstraZeneca e Sputnik V. As vacinas da Pfizer-BioNTech e Moderna são de RNA mensageiro (mRNA) e usam parte do código genético do vírus e induzem a produção de anticorpos direcionados para a proteína Spike[41]. A Pfizer-BioNTech, em estudo de fase 3 envolvendo mais de 44 mil voluntários, mostrou boa tolerabilidade e segurança, e uma eficácia de

38 Lin J-T et al. Safety and immunogenicity from a phase I trial of inactivated severe acute respiratory syndrome coronavirus vaccine. Antivir Ther [Internet]. 2007;12(7):1107–13.

39 Folegatti PM et al. Safety and immunogenicity of a candidate Middle East respiratory syndrome coronavirus viral-vectored vaccine: a dose-escalation, open-label, non-randomised, uncontrolled, phase 1 trial. Lancet Infect Dis [Internet]. 2020 Apr 20; 8

40 Thanh Le T et al. The COVID-19 vaccine development landscape. Nat Rev Drug Discov [Internet]. 2020 May 9;19(5):305–6.

41 Kaur SP, Gupta V. COVID-19 Vaccine: A comprehensive status report. Virus Res. 2020;288:198114.

95% após duas doses[42,43]. A Moderna, de forma semelhante, em estudo de fase 3 com 30 mil pessoas nos EUA, demonstrou bom perfil de segurança e eficácia de 94,5%[44].

A CoronaVac é uma vacina de vírus inativado desenvolvida pela Sinovac. Os resultados de um estudo pré-clínico mostraram que CoronaVac induziu boa produção de anticorpos neutralizantes em modelos animais e garantiu proteção individual parcial ou completa contra pneumonia intersticial grave em macacos. Um ensaio clínico de fase 1/2 da CoronaVac em participantes com idade entre 18–59 anos mostrou que CoronaVac foi bem tolerada e induziu boa resposta humoral contra SARS-CoV-2[45]. A CoronaVac também foi bem tolerada e imunogênica em adultos saudáveis com 60 anos ou mais, e as respostas de anticorpos neutralizantes ao SARS-CoV-2 se mantiveram nesta população[46]. A vacina continua em estudo, e teve aprovação para uso emergencial no Brasil, Indonésia e Turquia. Dados preliminares de ensaios de fase 3 na Turquia e Indonésia mostraram que a eficácia da vacina foi 91,2% e 65,3%, respectivamente. Pesquisadores no Brasil divulgaram uma eficácia de 78% para casos de COVID-19 precisando assistência ambulatorial ou hospitalar (Score 3 ou superior na Escala OMS) e de 50,4%, contra todas as formas da doença, em estudo de fase III em profissionais de saúde da linha de frente[47].

---

[42] Pfizer and biontech announce vaccine candidate against COVID-19 achieved success in first interim analysis from phase 3 study. https://www.pfizer.com/news/press-release/press-release-detail/pfizer-and-biontech-announce-vaccine-candidate-against

[43] Painter EM et al. Demographic Characteristics of Persons Vaccinated During the First Month of the COVID-19 Vaccination Program - United States, December 14, 2020-January 14, 2021. MMWR. Morbidity and mortality weekly report, 70(5), 174–177.

[44] Wu K et al. mRNA-1273 vaccine induces neutralizing antibodies against spike mutants from global SARS-CoV-2 variants. bioRxiv [Preprint]. 2021 Jan 25:2021.01.25.427948. doi: 10.1101/2021.01.25.427948.

[45] Wu Z et al Safety, tolerability, and immunogenicity of an inactivated SARS-CoV-2 vaccine (CoronaVac) in healthy adults aged 60 years and older: a randomised, double-blind, placebo-controlled, phase 1/2 clinical trial. Lancet Infect Dis. 2021 Feb 3:S1473-3099(20)30987-7.

[46] Zhang Y et al. Safety, tolerability, and immunogenicity of an inactivated SARS-CoV-2 vaccine in healthy adults aged 18-59 years: a randomised, double-blind, placebo-controlled, phase 1/2 clinical trial. Lancet Infect Dis. 2021 Feb;21(2):181-192. doi: 10.1016/S1473-3099(20)30843-4.

[47] Palacios R et al. Double-Blind, Randomized, Placebo-Controlled Phase III Clinical Trial to Evaluate the Efficacy and Safety of treating Healthcare Professionals with the Adsorbed COVID-19 (Inactivated) Vaccine Manufactured by Sinovac - PROFISCOV: A structured summary of a study protocol for a randomised controlled trial. Trials. 2020 Oct 15;21(1):853.

As vacinas Oxford-AstraZeneca e Sputnik V são produzidas a partir de um vírus geneticamente modificado que causa o resfriado em chimpanzés. A análise conjunta de ensaios de fase 2/3 no Reino Unido e no Brasil, e dados de segurança de mais de 20.000 participantes inscritos em quatro ensaios clínicos no Reino Unido, Brasil e África do Sul, mostra boa tolerabilidade e 70,4% de eficácia da vacina Oxford-AstraZeneca, tendo protegido contra infecção por COVID-19, hospitalizações e gravidade. A vacina pode ser armazenada e distribuída a 2–8°C, tornando-a particularmente adequada para distribuição. Intervalos mais longos entre as doses (8 a 12 semanas) estão associados a uma maior eficácia da vacina. Uma análise interina da vacina Gam-COVID-Vac (Sputnik V) demonstrou uma eficácia de 91,6% contra COVID-19 e boa tolerabilidade em uma grande coorte de 16.501 habitantes [48].

As vacinas podem ter perda ou redução de eficácia devido ao surgimento de novas variantes. A efetividade da vacina da Oxford-AstraZeneca foi questionada na África do Sul depois que um estudo com 2.000 voluntários jovens saudáveis não encontrou proteção significativa contra a doença leve e moderada causada pela nova variante B.1.351.

Estudos preliminares não encontraram evidências de que a variante B.1.1.7 tenha qualquer impacto na eficácia da vacina Pfizer-BioNTech [49,50]. Contudo, recentemente, foram relatados resultados insuficientes em ensaios de soroneutralização [51]. Para a variante B.1.351, contudo, há algumas evidências que indicam que uma das mutações da proteína (E484K) pode afetar a neutralização por

[48] Logunov DY, Dolzhikova I V, Shcheblyakov D V, Tukhvatulin AI, Zubkova O V, Dzharullaeva AS, Zyryanov SK, Borisevich S V, Naroditsky BS, Gintsburg AL. Safety and efficacy of an rAd26 and rAd5 vector-based heterologous prime-boost COVID-19 vaccine: an interim analysis of a randomised controlled phase 3 trial in Russia. *Lancet*. 2021;397:671-681. doi:10.1016/S0140-6736(21)00234-8.

[49] Xie Xet al. Neutralization of N501Y mutant SARS-CoV-2 by BNT162b2 vaccine-elicited sera. bioRxiv [Preprint]. 2021 Jan 7:2021.01.07.425740. doi: 10.1101/2021.01.07.425740. PMID: 33442691; PMCID: PMC7805448.

[50] Shen X et al. SARS-CoV-2 variant B.1.1.7 is susceptible to neutralizing antibodies elicited by ancestral Spike vaccines. bioRxiv [Preprint]. 2021 Jan 29:2021.01.27.428516. doi: 10.1101/2021.01.27.428516. PMID: 33532764; PMCID: PMC7852228.

[51] Wang P et al. Increased Resistance of SARS-CoV-2 Variants B.1.351 and B.1.1.7 to Antibody Neutralization. bioRxiv [Preprint]. 2021 Jan 26:2021.01.25.428137.

alguns anticorpos policlonais e monoclonais[10,52,53,54], o que pode impactar negativamente na eficácia das vacinas atualmente disponíveis. A variante P.1 também contém um conjunto de mutações que podem afetar sua capacidade de ser reconhecida por anticorpos[55]. Há evidências que sugerem que algumas das mutações na variante P.1 podem afetar sua transmissibilidade, perfil antigênico e imunogenicidade natural ou por vacinação[56,57], o que poderia afetar a efetividade das vacinas utilizadas no Brasil (Oxford-AstraZeneca e CoronaC). Novas informações sobre as características virológicas, epidemiológicas e clínicas dessas variantes devem ser pesquisadas, assim como a capacidade de escape à imunidade induzida por vacinas ou por infecções naturais, com consequentes novas infecções ou reinfecções. A possibilidade de escape à imunidade induzida pela vacina é preocupante, já que uma vez tendo uma grande proporção da população tenha sido vacinada, haverá pressão imunológica que poderá favorecer a disseminação de tais variantes mutantes[24].

As formas de se identificar a efetividade de vida real de vacinas contra novas variantes encontram alguns desafios, entre eles: a dificuldade em se utilizar um grupo placebo, em tempos de pandemia; a circulação variável de cepas numa mesma localidade; a confirmação laboratorial de novas variantes apenas em casos graves; e estudos do tipo caso-controle, em que se admite que pacientes com ausência do teste têm um teste negativo. Assim, seguindo a mesma lógica das vacinas para influenza, em que a proteção contra doença leve não é tão alta, mas sim contra o aparecimento de casos graves, é possível estudar a efetividade vacinal comparando os riscos de hospitalização e a letalidade entre

---

[52] Jangra S et al. The E484K mutation in the SARS-CoV-2 spike protein reduces but does not abolish neutralizing activity of human convalescent and post-vaccination sera. medRxiv [Preprint]. 2021 Jan 29:2021.01.26.21250543.

[53] Tada T et al.Neutralization of viruses with European, South African, and United States SARS-CoV-2 variant spike proteins by convalescent sera and BNT162b2 mRNA vaccine-elicited antibodies. bioRxiv [Preprint]. 2021

[54] Weisblum Y et al. Escape from neutralizing antibodies by SARS-CoV-2 spike protein variants. bioRxiv [Preprint]. 2020 Jul 22:2020.07.21.214759. doi: 10.1101/2020.07.21.214759. Update in: Elife. 2020 Oct 28;9.

[55] Naveca F C.SARS-CoV-2 reinfection by the new variant of concern (VOC) P.1 in Amazonas, Brazil. Virological.https://virological.org/t/sars-cov-2-reinfection-by-the-new-variant-of-concern-voc-p-1-in-amazonas-brazil/596

[56] Xie X et al. Neutralization of SARS-CoV-2 spike 69/70 deletion, E484K and N501Y variants by BNT162b2 vaccine-elicited sera. Nat Med (2021).

[57] CDC. Emerging SARS-CoV-2 Variants. https://www.cdc.gov/coronavirus/2019-ncov/more/science-and-research/scientific-brief-emerging-variants.html

grupos de pessoas com e sem fatores de risco para o desenvolvimento de doença mais grave. Dessa foram, a efetividade do produto vacinal é mensurada pela redução do risco no grupo com fatores predisponentes para maior gravidade, tendo como comparador o grupo sem a presença de fatores de risco. Essa abordagem constitui uma alternativa metodologicamente viável e eticamente equilibrada na relação risco-benefício oferecida aos participantes de um estudo dessa natureza [58].

Entre as vacinas atualmente disponíveis para uso contra COVID-19, as que utilizam vírus total inativado possuem, em tese, menor probabilidade de ter sua eficácia diminuída em caso de surgimento de novas variantes, em função da diversidade de epítopos induzindo imunidade. Atualmente, a única vacina disponível no Brasil com essa característica é a vacina Coronavac, cuja aprovação para uso emergencial pela ANVISA se deu em 17 de janeiro de 2021.

2.2 Objetivos

2.2.1 Objetivo primário

Avaliar a efetividade de duas doses da vacina adsorvida CoronaVac (de vírus inativado) para prevenir quadros clínicos moderados e graves de COVID-19 em profissionais de segurança pública e educação de 18 a 49 anos com comorbidades associadas a quadros graves da doença.

2.2.2 Objetivos secundários

2.2.2.1 Avaliação da efetividade

Avaliar a efetividade de pelo **menos uma dose** da vacina adsorvida CoronaVac (de vírus inativado) para prevenir quadros clínicos **moderados e graves** de COVID-19, com confirmação virológica, em profissionais de 18 a 49 anos com comorbidades associadas a quadros graves da doença, a partir da segunda semana após a **primeira dose**.

---

[58] Gail MH, Pee D. Robustness of risk-based allocation of resources for disease prevention. *Stat Methods Med Res*. 2020;29(12):3511-3524. doi:10.1177/0962280220930055

Avaliar a efetividade da vacina adsorvida CoronaVac (de vírus inativado) para prevenir quadros clínicos **moderados e graves** de COVID-19, com confirmação virológica, em profissionais de 18 a 49 anos com comorbidades associadas a quadros graves da doença, a partir da segunda semana após a **segunda dose**.

Avaliar a efetividade da vacina adsorvida CoronaVac (de vírus inativado), para prevenir quadros clínicos **graves** de COVID-19, com confirmação virológica, em profissionais de 18 a 49 anos com comorbidades associadas a quadros graves da doença, a partir da segunda semana após a **segunda dose**.

2.2.2.2 Avaliação de Segurança

Descrever a frequência da necessidade de atendimento médico por eventos adversos solicitados e não solicitados até 7 dias após cada dose da vacina (D7 e D35). Para o escopo do estudo será considerado como atendimento médico qualquer modalidade consulta clínica (ex.: tele consulta, domiciliar, hospitalar etc.).

2.2.2.3 Avaliação da Imunogenicidade

a. Avaliar a resposta imune à vacinação em um subgrupo de participantes antes de cada vacinação e a cada 3 meses, até 12 meses de seguimento.
b. Avaliar a resposta imune à vacinação mediada por células em um subgrupo de participantes antes de cada vacinação e a cada 3 meses, até 12 meses de seguimento.
c. Avaliar a presença de anticorpos contra SARS-CoV-2 antes de cada vacinação e a cada 3 meses, até 12 meses de seguimento.
d. Avaliação da capacidade de neutralização dos anticorpos em participantes que não soroconverteram após sorologia D28, através do Teste de Neutralização Viral (VNT);
e. Avaliação para populações de Memória por imunofenotipagem celular;

2.2.3 *F. Avaliação do PBMC por genotipagem para participantes que não apresentaram a soroconversão após a vacina.Objetivos exploratórios*

a. Verificar a associação de condições médicas subjacentes, por exemplo, diabetes, obesidade, dislipidemia, hipertensão e doença cardiovascular, na efetividade da vacina.

b. Avaliar a resposta imune humoral e celular em casos incidentes de COVID-19 ocorridos no estudo, em um subgrupo de participantes.
c. Descrever a resposta imune humoral e celular até um ano de estudo, em um subgrupo de participantes;
d. Descrever a resposta imune humoral e celular em participantes com infecção prévia por SARS-CoV-2.
e. Avaliar a efetividade de duas doses da vacina em infecções reincidentes e sintomáticas por SARS-CoV-2 detectadas sorologicamente e virologicamente, duas semanas após a segunda vacinação em pessoas com infecção prévia documentada.
f. Avaliar a efetividade de duas doses da vacina em prevenir o óbito por todas as causas e por COVID-19, duas semanas após a segunda vacinação.
g. Identificar o surgimento de novas variantes do vírus SARS-CoV-2 no período de acompanhamento do estudo.
h. Explorar a efetividade da vacina em subgrupos de participantes infectados pelas diversas variantes do vírus SARS-CoV-2.

2.3 Desenho geral do estudo

Este é um estudo quase-experimental com alocação baseada em risco. Serão incluídos profissionais de segurança pública e professores de ensino médio e superior da rede estadual de ensino. Na avaliação inicial será avaliada a presença de comorbidades associadas ao **maior risco** de doença grave por COVID-19, conforme Plano Nacional de Operacionalização da Vacinação Contra a COVID-19. Aqueles que apresentarem pelo menos uma dessas comorbidades serão convidados a receber a vacina de estudo em duas doses, com intervalo de 28 dias (±7 dias). Os participantes com **baixo risco** (sem comorbidades como fator de risco para COVID-19 grave de acordo com o Plano Nacional de Operacionalização da Vacinação Contra a COVID-19), **não** receberão a vacina no âmbito do projeto de pesquisa. Todos os participantes serão acompanhados por 12 meses a partir da inclusão no estudo. Espera-se que a imunização reduza o risco de doença moderada a grave daqueles com comorbidades ao nível daqueles da mesma faixa etária que não apresentam essas comorbidades, mas tem risco ocupacional análogo.

3 MÉTODOS

3.1 CENTRO DE ESTUDO

O centro de estudo será a Fundação de Medicina Tropical Dr. Heitor Vieira Dourado (FMT-HVD), que atuará de forma integrada aos órgãos de saúde e sanitários locais permitindo a vacinação e o acompanhamento dos participantes e a obtenção dos dados, conforme descrito neste protocolo. Os dados de contato do centro de estudo encontram-se no Anexo B. A Escola Normal Superior, unidade da Universidade do Estado do Amazonas, localizada em Manaus, Amazonas, é o local de recrutamento dos participantes do estudo,

3.2 PARTICIPANTES

O estudo será realizado em Manaus, em população de maior risco de infecção por SARS-CoV-2, dedicada à segurança pública (Policiais Civis, Militares, Trabalhadores do Departamento de Polícia técnico científica - DPTC, do Departamento de Trânsito - DETRAM-AM, da Secretaria de Segurança Pública do Estado do Amazonas – SSP/AM e do Corpo de Bombeiros Militar do Amazonas – CBMAM) e trabalhadores ativos da educação pública e privada, lotados em Manaus, Amazonas. Participantes de ambos os sexos e com idade de 18 a 49 anos poderão ser incluídos no estudo. Pessoas acima de 50 anos de idade já estão contempladas em campanha de vacinação, em Manaus. Os critérios de elegibilidade (inclusão e exclusão) no estudo deverão ser verificados antes da administração de cada uma das duas doses do esquema de imunização. Os participantes deverão cumprir todos os critérios de inclusão e não se enquadrarem em nenhum dos critérios de exclusão, descritos a seguir:

3.2.1 CRITÉRIOS DE INCLUSÃO

a. Adultos entre 18 anos e 49 anos de idade;
b. Ter disponibilidade de ser acompanhado durante o tempo de seguimento definido no estudo, por meio de visitas presenciais, contatos telefônicos e/ou digitais.

3.2.2 CRITÉRIOS DE EXCLUSÃO

a. Vacinação prévia específica para COVID-19;

b. Diagnóstico de COVID-19 nos últimos 28 dias (*swab* nasal e orofaríngeo);
c. História de reação alérgica grave ou anafilaxia aos componentes da vacina de estudo;
d. Relato de febre nas 72 horas que antecedem à vacinação (a inclusão pode ser adiada até que o participante complete 72 horas sem febre);
e. Caso possível ou confirmado de COVID-19 no dia da vacinação (nesta situação, a vacinação pode ser adiada até que o participante complete 72 horas sem sintomas ou seja descartado diagnóstico);
f. Ter recebido vacina com vírus vivo atenuado nos últimos 28 dias ou vacina inativada nos últimos 14 dias que antecedem a sua inclusão no estudo, ou ainda ter imunização programada para os primeiros 28 dias após sua inclusão no estudo;
g. Qualquer outra condição que, na opinião do investigador principal ou do seu representante médico, possa colocar em risco a segurança ou os direitos de um participante em potencial ou que o impeça de cumprir com este protocolo.
h. Gravidez ou lactante

3.3 DESFECHOS

3.3.1 DESFECHOS PRIMÁRIOS

Efetividade da vacina adsorvida CoronaVac (de vírus inativado), que será definida como a incidência de casos clínicos moderados e graves de COVID-19 (intensidade 4 ou superior conforme a escala de progressão clínica da OMS), em indivíduos de 18 anos e 49 anos, a partir da segunda semana após a segunda dose, entre grupos da mesma faixa etária com risco ocupacional análogo, possuindo ou não comorbidades associadas a quadros graves de COVID-19.

3.3.2 DESFECHOS SECUNDÁRIOS

Os desfechos secundários de efetividade são:

- Densidade de incidência de casos clínicos moderados e graves de COVID-19 (intensidade 4 ou superior conforme a escala de progressão clínica da OMS) a partir da **primeira dose;**

- Densidade de incidência de casos clínicos moderados e graves de COVID-19 (intensidade 4 ou superior conforme a escala de progressão clínica da OMS) a partir da **segunda dose;**
- Densidade de incidência de casos clínicos **graves** de COVID-19 (intensidade 6 ou superior conforme a escala de progressão clínica da OMS) a partir da segunda dose;
- Mediana dos escores de progressão clínica entre os casos moderados e graves;
- Densidade de incidência de casos clínicos confirmados virologicamente como COVID-19;
- Densidade de incidência de óbitos confirmados como COVID-19;
- Densidade de incidência de internação hospitalar por qualquer causa;
- Densidade de mortalidade por qualquer causa.

Os desfechos secundários de segurança são:

- Frequência da eventos adversos associados a necessidade de atendimento médico até 7 dias após cada dose da vacina (D7 e D35).
- Os desfechos secundários de imunogenicidade são:
- Frequência de detecção de anticorpos, em um subgrupo de participantes, antes de cada vacinação e a cada 3 meses, até 12 meses de seguimento.
- Resposta imune mediada por células, em um subgrupo de participantes, antes de cada vacinação e a cada 3 meses, até 12 meses de seguimento.

3.3.3 Desfechos exploratórios

- Densidade de incidência de casos clínicos moderados e graves de COVID-19 em subgrupos com condições médicas subjacentes (diabetes, obesidade, dislipidemia, hipertensão e doença cardiovascular).
- Perfil da resposta imune humoral e celular em casos incidentes de COVID-19 ocorridos no estudo, em um subgrupo de participantes.
- Perfil da resposta imune humoral e celular até um ano do estudo, em um subgrupo de participantes.
- Perfil da resposta imune humoral e celular em participantes com infecção prévia por SARS-CoV-2.

- Infecções reincidentes e sintomáticas por SARS-CoV-2 detectadas sorologicamente e virologicamente, duas semanas após a segunda vacinação em pessoas com infecção prévia documentada.
- Casos de óbito por todas as causas e por COVID-19, duas semanas após a segunda vacinação.
- Detecção de novas variantes do vírus SARS-CoV-2 no período de acompanhamento do estudo, incluindo as já descritas ou novas variantes.
- Densidade de incidência de casos clínicos moderados e graves de COVID-19 (intensidade 4 ou superior conforme a escala de progressão clínica da OMS) e participantes infectados por novas variantes do vírus SARS-CoV-2.

3.4 Procedimentos

3.4.1 Vacina adsorvida Coronavac *(vírus inativado)*

A vacina contém o vírus SARS-CoV-2 inativado, hidróxido de alumínio, hidrogenofosfato dissódico, di-hidrogenofosfato de sódio e cloreto de sódio.

3.4.1.1.1 Dose/Administração/Armazenamento

A temperatura de armazenamento da vacina, como indicada no rótulo do frasco é de 2 a 8°C. A dose da vacina corresponde a 0,5 mL. O local de administração recomendado é o músculo deltoide por injeção intramuscular. O esquema de imunização é de duas doses com um intervalo de 28 dias (±7 dias).

3.4.1.1.2 Lotes da vacina

Neste estudo, será utilizado um único lote de produção de vacina, para uso específico nesta pesquisa.

3.4.1.1.3 Indicações de uso

A vacina adsorvida Coronavac (Sinovac - vírus inativado) está aprovada no Brasil pela ANVISA desde o dia 17 de janeiro de 2021, para uso emergencial, durante a pandemia.

3.5 Procedimentos para o participante

3.5.1 Acompanhamento dos participantes

Ao todo, cada participante será acompanhado por um período de um ano após a sua inclusão no estudo. A equipe do estudo manterá contato periódico com os participantes da pesquisa por telefone e meios eletrônicos. Complementarmente, os desfechos de interesse do estudo poderão ser coletados a partir do sistema de vigilância epidemiológica oficial, utilizando os bancos do Sistema de Informação do Programa Nacional de Imunização (SI-PNI), SIVEP-Gripe (que contém dados sobre hospitalização e óbito por síndrome respiratória aguda grave - SRAG), Sistema Municipal de Imunização/Covid (SMV/Covid) e o Sistema de Informação sobre Mortalidade (SIM).

Ainda que se inicie (I) a fase de vacinação dos profissionais de segurança e educação de acordo com o Plano Nacional de Operacionalização da Vacinação Contra a COVID-19, (II) a população do estudo seja submetida a outra vacina própria para novas variantes, ou (III) novos estudos mostrem efetividade reduzida de Coronavac para P.1, os participantes incluídos no estudo, e que receberam as duas doses da vacina Coronavac, continuarão sendo acompanhados pelo estudo de acordo com a tabela de procedimentos do protocolo. A interrupção da vacinação ocorrerá somente quando as situações "II" e "III" ocorrerem antes da segunda dose da vacina (Vac2). Ainda assim, estes participantes continuarão sendo acompanhados pelo estudo de acordo com a tabela de procedimentos.

O estudo poderá ser interrompido em caso de descumprimento, por parte do investigador, de uma obrigação fundamental, incluindo-se, entre outros, a violação do protocolo do estudo clínico, o descumprimento de leis e regulamentos cabíveis ou descumprimento das normas ICH relativas às Boas Práticas Clínicas.

3.5.2 Descrição dos procedimentos do estudo por visita

3.5.2.1 Procedimentos do estudo

O protocolo possui seis tipos de procedimentos com o participante de acordo com o andamento do estudo (triagem, visitas de vacinação, contato telefônico, visitas de retorno, visita de hospitalização e visita não-programada).

3.5.2.1.1 Triagem

Após a divulgação do início do recrutamento, os participantes em potencial podem acessar um formulário eletrônico de cadastro, que contém inicialmente o processo de consentimento – e assinatura do TCLE. Após o aceite, serão coletadas informações demográficas, antropométricas, condições médicas pré-existentes e solicitação de contatos (telefone, e-mail do participante e de pessoas próximas). Caso o potencial participante não tenha preenchido o formulário eletrônico de consentimento e de dados iniciais, a triagem será feita no momento da visita e consistirá nos procedimentos abaixo:

- apresentação do estudo ao potencial participante;
- realização do procedimento de consentimento informado e aplicação do Termo de Consentimento Livre e Esclarecido (TCLE);
- verificação dos critérios de elegibilidade;
- coleta de dados demográficos, antropométricos, condições médicas pré-existentes dados de contato e sinais vitais.

Caso o potencial participante já tenha preenchido o formulário eletrônico, a triagem consistirá na revisão desses dados e correção, quando aplicável. Uma vez elegível, o participante realizará os procedimentos da visita de vacinação Vac1. Caso exista uma condição temporária que constitua um critério de exclusão, o potencial participante poderá ser convocado novamente para a visita de inclusão quando o impedimento não mais existir. O participante será considerado incluído no estudo no momento em que for vacinado (D0).

3.5.2.1.2 Visita de vacinação (Vac 1 e Vac2)

As visitas de vacinação ocorrerão em duas etapas

a. coleta de amostra de sangue para realização de sorologia e armazenamento em biorrepositório;
b. vacinação.

Nota 1: A Vac2 deverá ocorrer 28 dias (±7 dias) após Vac1.

Nota 2: As visitas presenciais Vac1 e Vac2 são obrigatórias para todos os participantes.

Nota 3: A vacinação será realizada pela equipe de assistência municipal de saúde e o seguimento via contatos telefônicos, visitas presenciais e complementarmente por meio de consulta aos sistemas de vigilância epidemiológica oficiais, pela equipe de pesquisa.

3.5.2.1.3 Contato telefônico (Ct)

Os contatos telefônicos serão realizados por meio de ligação, mensagem ou e-mail, a critério da equipe do estudo e do participante que informará a equipe as formas de contato de sua preferência durante a primeira visita do estudo (Vac1). Os meios eletrônicos e telefônicos incluem, entre outros, envio de mensagens de texto ou áudio por telefone ou internet através de programa de computador, aplicativo para tablet ou telefone inteligente (smartphone).

Os contatos telefônicos com os participantes ocorrerão em três formatos:

1 Verificação de eventos adversos precoces (D7 e D35):

   a. Eventos adversos (EA) solicitados (atendimento médico) e não-solicitados (quaisquer que o participante relate) nos primeiros sete dias após a visita de vacinação (Vac1 e Vac2).

2 Verificação de desfechos clínicos da COVID-19:
   a. Os desfechos clínicos de principal interesse são sintomas de síndrome gripal, necessidade de atendimento médicos, uso de oxigênio suplementar, hospitalização e óbito.
   b. Estas ligações ocorrerão mensalmente a partir de D60 até D360 (vide tabela de procedimentos).
   c. Nestas ligações as informações de contato deverão ser atualizadas.

3 Agendamento de visitas:
   a. Estas serão realizadas em até 7 dias de antecedência à visita de retorno do estudo (D90, D180, D270, D360)

Nota 4: será aceita uma janela de ±7 dias para as ligações mensais.
Nota 5: Todo contato realizado, ou tentativa de contato com o participante, deverá ser registrado no formulário do estudo (REDCap).
Nota 6: Não será necessário efetuar contato nas semanas em que está programada uma vista ao centro de pesquisa.

3.5.2.1.4 Visitas de retorno (Vr)

As visitas de retorno (Vr) acontecerão trimestralmente (D90, D180, D270 e D360) após a visita Vac1.

3.5.2.1.4.1 Procedimentos:

- Coleta de sangue para estudos imunológicos.
- Processamento e armazenamento em biorrepositório no centro de pesquisa.

Nota: será aceita uma janela de ±14 dias para as visitas trimestrais presenciais.

3.5.2.1.5 Visita de Hospitalização (Vh)

Caso haja a informação sobre hospitalização do participante – via contato telefônico ou via *linkage* probabilístico periódico dos dados pessoais do participante com dados de sistema de informação do sistema de saúde – a equipe do estudo tentará resgatar dados via prontuário do hospital para caracterização por meio da escala de progressão clínica da OMS. Se o participante ainda estiver internado, e tiver dado consentimento para tal, amostras de *swab* nasal e orofaríngeos serão coletadas para estudos imunológicos e moleculares do vírus. Esta visita será considerada uma visita não-programada (VNP).

3.5.2.1.6 Visita não-programada (VNP)

Caso haja informação sobre participantes que apresentem sintomas gripais, a equipe do estudo oferecerá de forma gratuita e contínua durante a vigência do estudo, a disponibilização de testes sorológicos tipo *swab* nasal (teste de antígeno e RT-PCR) e entrevista para monitoramento dos casos sintomáticos SEM hospitalização.

Nota sobre o *linkage* probabilístico:

O procedimento de *linkage* probabilístico consiste em identificar registros em bases de dados que não possuem identificadores como códigos, CPF, cartão do SUS ou correlatos. Tal procedimento será realizado mensalmente.

A técnica refere-se ao processo de unir registros relacionados à mesma entidade de uma ou mais base de dados. Uma entidade pode ser interpretada neste caso como uma pessoa, onde se deseja agregar os dados, por exemplo, de adoecimento e óbito. Grande parte dos dados do mundo real são caracterizados por serem ruidosos, incompletos e por vezes incorretamente formatados. Portanto, uma etapa crucial em qualquer projeto de vinculação de registros é a limpeza e a padronização de dados. Uma técnica bastante utilizada para a limpeza e padronização dos dados é classificar valores semelhantes no mesmo bloco. Valores com sons ou escritas parecidas devem pertencer ao mesmo grupo. Por exemplo, quando se trata de nome de pessoa, a semelhança fonética pode ser obtida com funções de codificação fonética como o soundex.

Como pode ser observado, o codificador soundex distribui o mesmo código para nomes foneticamente semelhantes, e com isso, realiza a etapa de limpeza dos dados contra os ruídos e erros de preenchimento. Ao mesmo tempo que permite a padronização e limpeza do banco, a técnica é útil para a anonimização dos dados dos indivíduos no banco.

Para a agregação dos dados do projeto, serão utilizadas as variáveis nome do paciente, nome da mãe, data de nascimento e sexo. Com os nomes codificados e “limpos”, a próxima etapa será a agregação de outros dados disponíveis para a realização do “record linkage”. Por exemplo, a junção do código

soundex com a data de nascimento e o sexo pode ser a combinação de dados passados para o "record linkage".

3.5.2.1.7 Captura de Eventos Adversos

Os eventos adversos serão capturados de forma solicitada e não-solicitada até 7 dias após a primeira e segunda dose da vacina. A equipe responsável pelo contato telefônico fará tal procedimento em D7 e D35 (sete dias após primeira e segunda dose respectivamente) com a intenção de captar sintomas e desconfortos durante o período de forma retrospectiva. Além disso, os participantes serão instruídos no dia da vacinação para entrar em contato com a mesma equipe, por contato telefônico ou por meio eletrônico (através do preenchimento de um formulário), no caso de algum desconforto neste período. Embora o estudo não tenha sido desenhado para avaliação de segurança, novas informações podem surgir. Desta forma, os eventos adversos devem ser notificados ao Instituto Butantan em até 24 horas depois do conhecimento da equipe através do e-mail *farmacovigilancia@butantan.gov.br*.

Classificação de eventos adversos:

Quanto à gravidade:

a. Evento adverso grave (EAG)

Qualquer evento clinicamente relevante que:

i. Requeira hospitalização.
ii. Possa comprometer o paciente, ou seja, que ocasione risco de morte e que exija intervenção clínica imediata para evitar o óbito.
iii. Cause disfunção significativa e/ou incapacidade permanente.
iv. Resulte em anomalia congênita.
v. Ocasione o óbito.

b. Evento adverso (EA)

Qualquer outro evento que não esteja incluído nos critérios de evento adverso grave (EAG). Os eventos não graves não representam risco potencial para a saúde do vacinado, embora também devam ser cuidadosamente monitorados, pois podem sinalizar um problema potencialmente maior

em relação à vacina ou à imunização, ou ter um impacto sobre a aceitabilidade da imunização em geral.

Quanto à causalidade:

De acordo com o Manual de Vigilância de Eventos Adversos Pós-Vacinação (2020), na vigência de informações incompletas ou insuficientes, um EA pós-vacina pode ser considerado inclassificável e pode também ser classificado como indeterminado, devido à falta de evidências, presença de dados conflitantes ou, ainda, inconsistência de associação causal e imunização. Desta forma, os eventos adversos do estudo não serão classificados quanto à causalidade.

Nota 7: Ainda assim, conforme o mesmo Manual, as notificações/investigações de EA pós-vacina são de extrema importância, porque em algum momento podem ser consideradas como um sinal, podendo gerar hipóteses de associação causal entre uma vacina e um determinado evento e propor a realização de estudos específicos destinados a testar uma associação causal.

Nota 8: Serão considerados eventos adversos apenas alterações e sintomas reportados até 35 dias depois da inclusão do participante no estudo (7 dias após segunda dose da vacina). Os desfechos do estudo – hospitalização, uso de oxigênio suplementar, óbito – não serão considerados eventos adversos e não seguirão o fluxo de notificação como tal.

3.5.3 Procedimento em caso de perda de visita ou contato

O participante será considerado perda de seguimento caso não responda a mais de três contatos telefônicos consecutivos e não compareça a uma visita de retorno após as visitas de vacinação. O status do participante deverá ser alterado para “perda de seguimento” e registrado.

Será Considerada visita não-realizada:

- Caso não seja possível contato com o participante após 3 (três) dias consecutivos, em horários diferentes
- Caso o participante não compareça à visita agendada por 2 (duas) vezes.

Será considerada perda de seguimento quando:

- participante não realizar 2 (duas) visitas consecutivas (seja telefônica ou presencial).

3.6 Tabela de procedimentos do estudo

O período de permanência no estudo para cada participante será de aproximadamente um ano após a vacinação, que será considerado como Dia 0 do estudo (visita Vac1). Durante o estudo são previstas visitas presenciais e contatos para o acompanhamento de cada participante (Tabela 1).

3.6.1 Teste de Neutralização Viral (VNT)

Para realizar o teste de neutralização viral (VNT), células Vero CCL-81 serão distribuídas em placas transparentes de 96 poços (10 4 células por poço) no dia anterior. Amostras de soro provenientes de pacientes vacinados com a vacina Coronavac (28 dias após a primeira dose e 60 dias após a segunda dose) serão incubadas à 56ºC, por 30 minutos, para inativação de quaisquer componentes do soro que possam influenciar na neutralização. Para avaliar a capacidade de neutralização dos anticorpos, as amostra

s de soro inativadas serão diluídas, de forma seriada, em meio de cultivo DMEM suplementado com 100 U/ml de penicilina, 100 µg/ml de estreptomicina e 2,5% de soro fetal bovino.

Para cada soro, oito diluições serão testadas (1:20, 1:40, 1:80, 1:160, 1:320, 1:640, 1:1280 e 1:2480) em triplicata. As amostras diluídas serão incubadas com o SARS-CoV-2 na concentração final de 10^3 TCID50/ml, à 37ºC, por 1h. Após esse período, as amostras diluídas, contendo o vírus, serão distribuídas na placa de células Vero CCL-81 e mantidas em estufa umidificada à 37°C e 5% de CO 2, por 72h. Após isso, o meio de cultivo contendo soro e vírus será descartado, as células serão fixadas com formol 10%, por 20 min, e coradas com cristal violeta para analisar a capacidade de neutralização a partir da presença ou ausência de efeito citopático nas réplicas de cada diluição.

3.6.2 Imunofenotipagem celular para Populações de Memória

As amostras de sangue periférico de cada paciente serão utilizadas para a realização da imunofenotipagem celular, por meio da técnica de Citometria de Fluxo (CF). As subpopulações de células T e B de memória serão marcadas com anticorpos monoclonais conjugados a fluorocromos (Quadro 01) que se ligam a marcadores de superfície específicos (clusters of differentiation) presentes nas populações de interesse. A caracterização imunofenotípica será realizada pelo Citômetro de Fluxo (BD FACSCanto II).

Neste estudo será utilizado um painel de anticorpos monoclonais marcados, divididos em 03 tubos para a quantificação e caracterização das populações celulares do estudo, como descrito abaixo.

Tubo	Anticorpo	Fluorocromo	Perfil celular
01	-	-	Controle Negativo
02	Human Anti-CD4	FITC	Linf. T
	Human Anti-CD8	PE	
	Human Anti-CD45RA	PE-Cy7	
	Human Anti-CD3	PerCP	
	Human Anti-CD197/CCR7	APC	
	Human Anti-CD27	APC-Cy7	
03	Human Anti-IgG	FITC	Linf. B
	Human Anti-CD19	PE	
	Human Anti-HLA-DR	PE-Cy7	
	Human Anti-CD38	PerCP	
	Human Anti-IgM	APC	
	Human Anti-CD27	APC-Cy7	

Quadro 1. Descrição dos tubos e perfil celular dos anticorpos monoclonais que serão utilizados na citometria de fluxo.

3.6.3 *TESTE DE ANTÍGENO*

O Panbio COVID-19 Ag Rapid Test Device é um teste rápido de diagnóstico in vitro para a detecção qualitativa do antígeno SARS-CoV-2 (Ag) em amostras nasofaríngeas de indivíduos que atendem aos critérios clínicos e/ou epidemiológicos da COVID-19.[59] [60]

3.6.3.1 Coleta do swab nasal

Deve-se inclinar a cabeça do paciente ligeiramente para trás, cerca de 45°-70°, para endireitar a passagem da frente do nariz. O *swab* deve atingir uma profundidade igual a distância das narinas até a abertura externa da orelha. A coleta deve ocorrer somente em uma narina. Será realizado teste rápido de antígenos COVID-19 Rapid Test Device.

Panbio COVID-19 Ag Rapid Test Device será realizado imediatamente após a coleta seguindo as instruções do fabricante (leitura em 15 min)[61].

3.6.4 RT-PCR diagnóstico de Covid-19

3.6.4.1 Coleta dos swabs nasal e orofaríngeo

Introduzir um *swab* pela narina, paralelamente ao palato, até a nasofaringe, realizar movimentos rotatórios para captação de células da nasofaringe, e absorção da secreção respiratória. Realizar o mesmo procedimento com outro *swab* na outra narina. O terceiro *swab* será utilizado na coleta de secreção respiratória da parte posterior da orofaringe evitando contato com a língua, dentes e bochechas para minimizar contaminação.

Os três *swabs* deverão ser acondicionados em um único tubo de rosca estéril, contendo o meio de transporte viral (líquido rosa). Após inserir os *swab*s no tubo, caso as hastes não sejam flexíveis e não

---

[59] Stephen M, Hahn M.D. Coronavírus (COVID-19) Update: FDA Authorizes First Antigen Test to Help in the Rapid Detection of the Virus that Causes COVID-19 in Patients. Commisioner of Food and Drugs, 2020 May 09

[60] Sandeep Kumar Vashist In Vitro Diagnostic Assays for COVID-19: Recent Advances and Emerging Trends, 2020 April 05

[61] Eliseo Albert, Ignacio Torres, Felipe Bueno, Dixie Huntley, Estefanía Molla, Miguel Angel Fernandez-Fuentes et al. Field evaluation of a rapid antigen test (Panbio™ COVID-19 Ag Rapid Test Device) for COVID-19 diagnosis in primary healthcare centres Research Note Vol 27, Issue 3, P472.E7-472.E10, March 01, 2021

quebrem facilmente, cortá-las com tesoura e tampar o tubo. Colocar o tubo num saco plástico dentro do isopor com gelox e transportar na posição vertical para garantir que o *swab* fique imerso na solução.[62]

3.6.4.2 Detecção de SARS-CoV-2 P.1 / VOCs por RT – PCR

Será realizado RT-PCR para a detecção de VOCs que usam um iniciador direto (P.1/VOCs-FNF 5′-GGGTGATGCGTATTATGACATGGTTGG), um iniciador reverso (P.1/VOCs-FNR 5′-CTAGCACCATCATCATACACAGTTCTTGC) e uma sonda (P.1/VOCs-FNP 5′ FAM (ZEN) TGGTTGATACTAGTTTGAAGCTAAAA) para detectar a deleção ORF1b (NSP6: S106del, G107del, F108del) encontrada nos três VOCs (P.1, B.1.1.7 e B.1.351). Ambos os primers serão usados a 300 nM e a sonda a 150 nM (concentração final), com TaqMan One-step Fast Virus master Mix (ThermoFisher Scientific, no. 4444434). Todos os dados de RT-PCR em tempo real coletados neste experimento serão adquiridos usando o QuantStudio 5 Real-Time PCR System e o software de design e análise QuantStudio v.1.4.1 (ThermoFisher Scientific) (5).

3.6.4.3 Amplificação e sequenciamento de SARS-CoV-2

As amostras positivas na PCR serão submetidas à amplificação e sequenciamento de próxima geração. Resumidamente, os RNAs serão extraídos com o kit de purificação de ácido nucléico total Maxwell RSC Viral e depois convertidos em DNA complementar com transcriptase reversa Superscript IV. Os amplicons serão amplificados com SuperFi II Green PCR master mix (uma DNA polimerase de revisão com fidelidade> 300 × Taq da ThermoFisher Scientific, precipitados com PEG 8000 e quantificados usando um fluorímetro. Amplicons agrupados normalizados de cada amostra serão usados para preparar bibliotecas de sequenciamento de próxima geração com Nextera XT e agrupados com 500 ciclos de MiSeq Reagent Kit v.2 em 2 × 250 ciclos ou 2 × 150 ciclos de execuções

[62] Diretrizes Laboratoriais para o Diagnóstico e Detecção de Infecção pelo Novo Coronavírus (2019-nCoV). OPAS/BRA/nCov/20.009 01 de fevereiro de 2020

de extremidades emparelhadas. Todos os dados de sequenciamento serão coletados usando a plataforma de sequenciamento MiSeq e o software Miseq Control v.2.6.2.1 (Illumina)[63]

3.6.5 Testes laboratoriais complementares

Para medir o reconhecimento de variantes pelos anticorpos induzidos pela vacina, os níveis destes anticorpos e a amplitude ("breadth") dos mesmos contra fragmentos de proteínas da linhagem selvagem e variantes da proteína Spike, serão realizados ensaios Luminex de alto rendimento em indivíduos após vacinação e/ou exposição ao SARS-CoV-2. Anticorpos para proteínas não-S (por exemplo, terminal N) serão usados para distinguir a vacina das respostas naturais à infecção.

Para avaliar o escape de neutralização de anticorpos de VoC, mediremos a capacidade de neutralização de plasma/soros de indivíduos convalescentes, vacinados e aqueles com imunidade híbrida (vacinados mais infectados) contra diferentes variantes da proteína Spike usando pseudovírus para SARS-CoV-2, com base no sistema de empacotamento VSV e expressão em células humanas (HEK) expressando o receptor hACE2.

Para caracterizar a duração das respostas imunes e correlatos de proteção, agruparemos as avaliações ao longo do tempo, obtendo níveis de anticorpos em amostras repetidas.

[63] Ref Naveca, F.G., Nascimento, V., de Souza, V.C. *et al.* COVID-19 in Amazonas, Brazil, was driven by the persistence of endemic lineages and P.1 emergence. *Nat Med* **27**, 1230–1238 (2021). https://doi.org/10.1038/s41591-021-01378-7

Tabela 1. Tabela de procedimentos do estudo.

	D0	D7	D28[3]	D35[11]	D60	D90[4]	D120	D150	D180[4]	D210	D240	D270[4]	D300	D330	D360[4]	Vh	VNP
Tipo de visita	Vac1[9]	Ct	Vac2	Ct	Ct	Vr/3m	Ct	Ct	Vr/6m	Ct	Ct	Vr/9m	Ct	Ct	Vr/12m	VNP	VNP
Visita nº	1	2	3	3a	4	5	6	7	8	9	10	11	12	13	14	15	-
Janela de visita	0	±1	±7	± 1	±7	±7	±7	±7	±14	±7	± 7	±14	±7	±7	± 1	-	-
Triagem [1]	X																
Consentimento livre e esclarecido	X																
Dados demográficos e histórico médico	X																
Sinais vitais [2]	X		X														
Teste de gravidez[10]	X		X														
Revisão dos critérios de inclusão/exclusão	X																
Coleta de sangue para sorologia	X		X			X			X			X			X		
Coleta de *swab* nasal/ orofaríngeo (RT-PCR)																X	X
Coleta de sangue para armazenamento	X		X			X			X			X			X	X	X
Vacinação	X		X														
Contato telefônico		X[5]		X[5]	X[6]	X[7]	X[6]	X[6]	X[7]	X[6]	X[6]	X[7]	X[6]	X[6]	X[7]		
Coleta de dados clínicos e laboratoriais																X[8]	X

Legenda:

Vac1: visita de vacinação 1; Ct: contato telefônico; Vr: visita de retorno da vacinação; Vh: visita de hospitalização; VNP: visita não-programada

[1] Processo de seleção de potenciais participantes

[2] Pressão arterial, frequência cardíaca e glicemia capilar

[3] Segunda dose da vacina (Vac2) com janela de ±7 dias

[4] Visitas presenciais para coleta de sangue com janela de ±7 dias

[5] Verificação de eventos adversos precoces

[6] Contato telefônico (ligação ou mensagem) com janela de ± 7 dias

[7] Contatos realizados em até 7 dias de antecedência à visita de retorno do estudo (D90) e ±14 dias de retorno do estudo nas visitas D180, D270 e D360.

[8] Caso participante seja hospitalizado

[9]Participantes que não tomarem a vacina não retornarão para visita de D28 e nem serão contatados em D7 e D35

[10] Teste de gravidez na urina pra mulher em idade fértil na triagem

[11] A janela desta visita será ampliada para ±7 dias em virtude da possibilidade de antecipação da segunda dose da vacina também em 7 dias.

3.7 Critérios e procedimentos para exclusão do participante

A qualquer momento o participante pode ser excluído do estudo, se assim desejar ou se o médico/investigador do estudo considerar necessário que um participante seja excluído em prol de sua segurança e bem-estar. O participante não sofrerá nenhum tipo de prejuízo caso opte por sair do estudo. O status do participante será alterado para “excluído”.

Será considerado **Desistente** quando:

- participante informar ao call center que não deseja mais participar do estudo e confirmar esse desejo para a equipe da ouvidoria.

Será considerado **Excluído** quando:

- médico/investigador do estudo considerar necessário que seja excluído em prol de sua segurança e bem-estar. O participante não sofrerá nenhum tipo de prejuízo caso opte por sair do estudo. O status do participante será alterado para “excluído”.

Nota 9: Caso a exclusão ocorra antes da segunda dose da vacina, o participante será orientado a retornar para a vacinação mesmo que recuse coleta de sangue para o estudo. Essa informação deve ser registrada pela equipe do estudo.

3.8 Tamanho amostral

3.8.1 *Cálculo de tamanho amostral e poder estatístico*

O tamanho amostral está direcionado a acumular suficientes casos para análises de efetividade. As hipóteses nulas (H_0) e alternativa (H_1) para a análise primária de casos clínicos moderados e graves de COVID-19 (intensidade 4 ou superior conforme a escala de progressão clínica da OMS) nos grupos com vacinação antecipada e vacinados pelo calendário do Ministério da Saúde são:

H_0: A incidência de casos clínicos moderados e graves de COVID-19 (intensidade 4 ou superior conforme a escala de progressão clínica da OMS) em participantes portadores de comorbidades tem intervalo de confiança de 95% igual a do grupo sem comorbidades.

H_1: A incidência de casos clínicos moderados e graves de COVID-19 (intensidade 4 ou superior conforme a escala de progressão clínica da OMS) em participantes portadores de comorbidades tem intervalo de confiança de 95% maior ao do grupo sem comorbidades.

As seguintes premissas e limites foram utilizadas para obter o tamanho amostral:

Incidência de hospitalização na população geral*	0,41%
Incidência de hospitalização em portadores de comorbidades*	1,03%
Alfa unicaudal	0,5%
Poder	90%
Taxa de perda anual	30%
Número de análises interinas	1
Recrutamento de participantes	Uniforme durante a duração da inclusão
Razão de alocação	1:1
Tempo desejado para análise primária	6 meses

* Dados obtidos a partir do SIVEP-Gripe e IBGE.

O desenho requer que **10.156** participantes sejam incluídos na proporção de 1:1 (**5.078 com alto risco e 5.078 de baixo risco para COVID-19 grave**). A população a ser vacinada será composta por indivíduos com alto risco para COVID-19 grave (**5.078**), enquanto a população de baixo risco não será contemplada com a vacina no âmbito deste projeto, sendo apenas acompanhada por 12 meses conforme tabela de procedimentos. A participação neste projeto não será um fator restritivo à

vacinação de acordo com o Plano Nacional de Operacionalização da vacinação contra a COVID-19 para os indivíduos com baixo risco para COVID-19 grave. Se, após os 12 meses, o participante do grupo de baixo risco (sem vacina), não tiver recebido CoronaVac, ou qualquer outra vacina disponível, o mesmo receberá a vacina, exatamente como oferecida no outro grupo (duas doses de CoronaVac com intervalo de 28 dias – podendo ser 7 dias antes ou depois).

3.9 POPULAÇÃO FONTE DO ESTUDO E ÁREA DE RECRUTAMENTO

A população do estudo será recrutada entre trabalhadores do sistema de segurança pública e do sistema de educação público ou privado, lotados em Manaus, com idade entre 18 e 49 anos. Seguem populações estimadas potencialmente elegíveis:

Policiais civis: **1.217**

Policiais militares: **6.744**

Corpo de bombeiros: **651**

Profissionais de ensino médio da rede estadual de educação: **6.335**

Profissionais da Universidade do Estado do Amazonas: **774**

Profissionais da rede privada de ensino: **10.163**

Total: **25.884 potenciais inclusões**

3.10 COLETA E GERENCIAMENTO DE DADOS

Este estudo utilizará o REDCap para a captura de dados e o mesmo será utilizado como documento fonte das informações contidas nele, uma vez que os dados coletados serão inseridos diretamente na plataforma de dados.

3.11 PROGRAMA DE ANÁLISE E PROCESSAMENTO DE DADOS

O REDCap, que será utilizado para a captura de dados, é uma plataforma validada e utilizada por diversas instituições no mundo todo. No caso do estudo, será utilizado o REDCap instalado

localmente no servidor da FMT-HVD. Backups periódicos são realizados pela equipe da FMT-HVD para garantir a segurança, rastreabilidade e completude dos dados.

3.12 Plano de Análise

De forma geral, as variáveis qualitativas associadas a cada desfecho serão apresentadas de forma descritiva com medidas absolutas (n) e relativas (%). As variáveis quantitativas, poderão ser apresentadas com medidas tais como média, desvio padrão, mediana, valores mínimo e máximo e, eventualmente, intervalo de confiança de 95% para a média. Para testes estatísticos e intervalos de confiança será utilizado um valor de significância de 5% bicaudal. O plano de análise foi feito de acordo com os desfechos de efetividade, segurança e imunogenicidade. Na análise final, o critério de sucesso será atingido (p>0,05), caso o Risco Relativo estimado seja igual entre os grupos, comprovando a efetividade vacinal.

3.12.1 Efetividade

Será calculada a incidência de casos moderados e graves de COVID-19 entre os dois grupos da mesma faixa etária. Serão comparadas as incidências nos dois grupos mediante a estimação do Risco Relativo (RR).

O efeito de covariáveis na incidência será considerado, mediante o ajuste de um modelo de regressão linear generalizada log-binomial. Considerando o número de internações hospitalares e óbitos em escala de tempo em dias, será feita uma análise descritiva que inclui curvas de sobrevivência de Kaplan-Meier e funções de taxa de falha comparadas mediante o teste de log-rank por grupos de risco. Será calculado o Hazard Ratio (HR) usando regressão Cox de riscos proporcionais por grupos de risco. Os escores de progressão clínica entre os casos moderados e graves de ambos os grupos, serão comparados com base na modelagem de efeitos mistos não lineares. A estimativa nesses modelos é feita usando estimativa de máxima verossimilhança.

A análise primária de efetividade será uma análise modificada por protocolo calculada com todos os casos confirmados virologicamente de COVID-19 internados ou que evoluírem a óbito que ocorrerem

no período de 1 ano após a aplicação da primeira dose da vacina. Essa análise será realizada na população Por Protocolo, como análise primaria. Na população por Intenção de Tratar será considerada como análise secundária

3.12.2 Segurança

A análise de segurança será apresentada com medidas descritivas absolutas (n), relativas (%) e intervalos de confiança para 95% para necessidade de atendimento médico. Análises adicionais como frequência de sintomas e avaliação de segurança pode ser realizadas, de acordo com a completude dos dados coletados.

3.12.3 Imunogenicidade

A imunidade inicial (presença / ausência de anticorpos anti-SARS-CoV-2, incluindo o título médio geométrica [GMT]) será avaliada entre todos os participantes que contribuíram com uma amostra.
Serão calculadas médias geométricas dos títulos de anticorpos geral e discriminada por algumas variáveis de interesse. Uma análise marginal será feita para determinar as variáveis, qualitativas e quantitativas, associadas à média geométrica dos títulos de anticorpos, serão usados teste t de Student, ANOVA e coeficiente de correlação de Pearson. Um modelo de regressão linear múltiplo será ajustado para quantificar o efeito das diversas variáveis no título de anticorpos. O modelo final proposto será escolhido conforme uma análise de diagnóstico.

A análise irá descrever os resultados de imunogenicidade de um subgrupo de participantes em termos de soroconversão de anticorpos neutralizantes. As médias geométricas dos títulos também serão descritos entre aqueles com soroconversão e serão comparados entre aqueles que adquiriram a infecção e um subgrupo daqueles sem infecção. As análises consideraram a presença de títulos de anticorpos antes da primeira vacinação e o relato documentado de infecção prévia. As seguintes estatísticas descritivas serão calculadas para cada avaliação e cada grupo:

- Taxas de soroconversão com intervalos de confiança 95% comparando contra o título basal

- Mediana dos títulos com intervalos de confiança 95%
- Gráficos da distribuição cumulativa reversa de títulos / concentrações
- Gráficos de caixas das distribuições de títulos / concentrações

Os dados basais demográficos, antropométricos e de exposição prévia a SARS-CoV-2 serão apresentados de forma descritiva. Em relação a raça ou origem étnica, será considerada a autodeclaração do participante. As análises poderão ser realizadas conforme faixa etária e grupo, usando o Teste Qui-quadrado ou Exato de Fisher para variáveis binárias, quando apropriado e análise de variância (ANOVA) para as variáveis contínuas.

A distribuição dos participantes incluindo os números de pessoas incluídas, triadas e vacinadas será resumida e apresentada no diagrama CONSORT, incluindo descontinuação do estudo. As causas de falha na triagem e descontinuação serão descritas.

3.12.4 Gerenciamento de dados faltantes/ausentes

Não serão imputados dados para a análise primária de eficácia por protocolo. Portanto, as análises excluirão participantes com dados faltantes ou não avaliáveis. Se houver uma quantidade de dados faltante excessiva ou houver algum tipo de padrão entre os dados faltantes, ferramentas estatísticas adicionais podem ser implementadas.

3.13 Monitoramento de dados do estudo

3.13.1 Comitê de monitoramento de dados e segurança

Este estudo terá um Comitê de Monitoramento de Dados e Segurança (CMDS) constituído com base nas recomendações do Ministério de Saúde[64]. Este comitê será nomeado pelos investigadores proponentes e estará composto por três pessoas com uma das seguintes qualificações:

- Médico Infectologista: deve ter experiência na realização de estudos relacionados com o objeto do estudo.
- Epidemiologista: deve ter experiência na realização de estudos ou em vigilância epidemiológica relacionados com objeto do estudo.
- Estatístico: Deve ter graduação ou pós-graduação em estatística. Deve ter experiência em análise de dados na área da saúde.
- Outros profissionais da área da saúde: devem ter experiência no desenho, condução ou na análise de ensaios clínicos.

Os membros do Comitê (anexo B) serão totalmente independentes dos investigadores proponentes e do patrocinador. Não há previsão de pagamento de honorários para os membros do comitê. Membros da equipe do estudo poderão dar apoio às atividades do comitê, mas não poderão fazer parte do mesmo.

O Comitê poderá fazer recomendações por escrito para continuar, modificar, suspender ou encerrar o estudo. Essas recomendações serão recebidas pelo investigador, que poderá discordar das recomendações através de resposta justificada que será comunicada da mesma forma. Todas as decisões devem ser encaminhadas para o sistema CEP/CONEP.

3.13.2 Análises e revisão interina

[64] Brasil M da S. Diretrizes Operacionais para o Estabelecimento e o Funcionamento de Comitês de Monitoramento de Dados e de Segurança. 2008

Análises interinas poderão ser feitas quando (1) 10%, (2) 25% e (3) 50% dos participantes completarem a visita D60. É possível que o CMDS solicite uma análise interina a qualquer momento, desde que devidamente justificada e que a solicitação seja aceita por parte do investigador principal.

3.13.3 Definições de caso de COVID-19

A definição de vigilância de caso para a doença causada pelo SARS-CoV-2 (COVID-19) que será utilizada neste estudo será aquela declarada pelos guias de FDA[65].

3.14 Garantia/controle de qualidade de dados

3.14.1 Monitoramento clínico

O estudo contará com uma equipe de controle de qualidade interno, que verificará periodicamente o preenchimento dos formulários e do termo de consentimento.

3.14.2 Acesso aos documentos fonte

Este estudo utilizará o REDCap (Research Electronic Data Capture) para a captura de dados e o mesmo será utilizado como documento fonte das informações contidas nele. Desta forma, caso haja necessidade de auditoria ou inspeção, os dados serão disponibilizados para visualização. Os dados identificáveis do participante terão acesso restrito a profissionais da equipe que necessitem dessa informação.

[65] Development and Licensure of Vaccines to Prevent COVID-19: Guidance for Industry [Internet]. Silver Spring; 2020. Available from: www.fda.gov/media/139638/download

4 ÉTICA E PUBLICAÇÕES

4.1 APROVAÇÕES PARA A REALIZAÇÃO DA PESQUISA

4.1.1 DECLARAÇÃO DO MARCO REGULATÓRIO DO ESTUDO

O protocolo está regulado pelas Resoluções 466/12[66] e 441/2011[67] do Conselho Nacional de Saúde, a Declaração de Helsinque em sua versão mais recente e as Guias sobre Boas Práticas Clínicas da Conferência Internacional de Harmonização. Qualquer outra norma aplicável que venha ser incorporada à normatividade brasileira no decorrer do protocolo será aplicada a este estudo.

4.1.2 APROVAÇÕES DO ESTUDO

O investigador principal conduzirá o estudo em conformidade com o protocolo aprovado pelos órgãos ético-regulatórios.

4.2 EMENDAS AO PROTOCOLO

Emendas ao protocolo podem ser necessárias a depender do andamento do estudo e disponibilidade de novas informações na literatura. Toda alteração do protocolo deverá ser previamente aprovada pelo Comitê de Ética antes de ser implementada, a menos que esta alteração seja em prol da segurança do participante de pesquisa. O estudo está registrado em bases de dados públicas que serão atualizados assim que a emenda obtiver todas as aprovações necessárias.

4.3 CONSENTIMENTO LIVRE E ESCLARECIDO

4.3.1 IMPLEMENTAÇÃO E DOCUMENTAÇÃO DO CONSENTIMENTO LIVRE E ESCLARECIDO

---

[66] Brasil M da S, de Saúde CN. Resolução nº466, de 12 de dezembro de 2012. Diretrizes e normas regulamentadoras de pesquisas envolvendo seres humanos. Diário Of da União da República Fed do Bras. 2013;150(112).

[67] CNS/MS. Resolução CNS Nº 441 [Internet]. Brasília: Conselho Nacional de Saúde; 2011 [citado 2011 Set 29]. Disponível em: http://conselho.saude.gov.br/resolucoes/2011/Reso441.pdf.

O Termo de Consentimento Livre e Esclarecido deverá ser aprovado pelo Comitê de Ética em Pesquisa e a Comissão Nacional de Ética em Pesquisa, quando aplicável, antes da sua utilização. Neste estudo o processo de consentimento eletrônico será feito por meio da plataforma REDCap, já utilizada pela FMT-HVD em outros estudos.

A plataforma possui um módulo criado especialmente para esse processo e trata-se de uma plataforma com amplo uso em pesquisa clínica em todo mundo. Através de anúncios de recrutamento o potencial participante pode ter acesso ao link com o TCLE antes de estar no local do estudo. Neste link estará disponível o documento em pdf que pode ser baixado e lido com cautela o tempo que for necessário. Em caso de dúvidas, o documento possui o telefone para contato e o e-mail do estudo. O processo de consentimento também pode ser realizado no local do estudo, com algum membro da equipe do estudo.

O processo de consentimento é documentado pelo mesmo sistema através de perguntas que o participante precisa responder para garantir a compreensão do estudo. O TCLE é considerado assinado quando o participante clica no campo abaixo e em “enviar”.

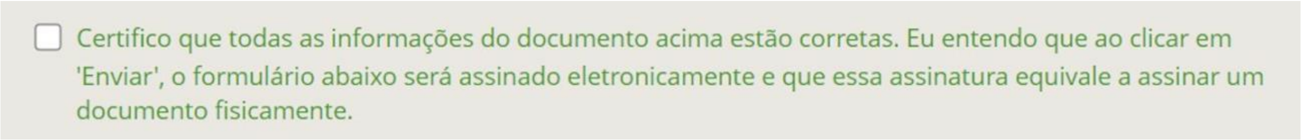

Uma via do documento é enviada via e-mail do participante e durante a visita de inclusão será enfatizada a importância de que ele guarde em seus arquivos esse documento eletrônico. A equipe do estudo – no momento do contato telefônico de D7 – confirmará que o participante recebeu sua via. Além disso, o próprio sistema envia uma notificação o administrador quando um e-mail é inexistente e ele pode notificar à equipe do contato telefônico para buscar um novo e-mail. No caso de instabilidade, ou falha, no sistema eletrônico adotado, o processo será realizado presencialmente e com documentos impressos.

O participante poderá retirar seu consentimento a qualquer momento, mesmo após o início dos procedimentos do estudo, sem prejuízo para seu atendimento na instituição, caso este atendimento

seja decorrente da sua participação no estudo. Os direitos e o bem-estar dos participantes serão protegidos enfatizando-se que a qualidade do seu atendimento médico não será afetada caso ele não aceite participar no estudo.

4.3.2 *AMOSTRAS BIOLÓGICAS*

4.3.2.1 Local de realização dos testes previstos no protocolo

Alíquotas de sangue dos participantes serão enviadas periodicamente para o Instituto Butantan, em São Paulo, que será responsável pela realização dos testes sorológicos. Os demais testes contidos neste protocolo serão realizados na FMT-HVD.

4.3.2.2 Armazenamento de amostras biológicas e uso de biorrepositório

Todo o material biológico coletado no decorrer do estudo, com a permissão do participante, será guardado para a possível utilização em pesquisas futuras em biorrepositório específico do estudo, localizado na Unidade de Pesquisa Clínica Carlos Borborema, na Fundação de Medicina Tropical Dr. Heitor Vieira Dourado (UPCCB/FMT-HVD), em Manaus-AM, por um período de até 10 anos conforme Resolução CNS 441/2011. Qualquer novo projeto de pesquisa que seja realizado no futuro com o material armazenado será submetido para aprovação da Comissão de Ética em Pesquisa. Todos os procedimentos de manejo do biorrepositório seguirão as orientações da Resolução N° 441 do Conselho Nacional de Saúde[68].

Testes complementares serão realizados com as amostras biológicas armazenadas em biorrepositorio, as quais serão enviadas ao ISGlobal, em Barcelona, Espanha. As amostras que serão enviadas não contêm nenhum material genético viável dos participantes.

A caracterização extensa da resposta imunológica gerada pela vacina é fundamental para uma compreensão adequada do seu perfil de segurança e imunogenicidade. O armazenamento de amostras de sangue e *swab* nasal dos participantes incluídos neste estudo irá possibilitar a realização

[68] CNS/MS. Resolução CNS Nº 441 [Internet]. Brasília: Conselho Nacional de Saúde; 2011 [citado 2011 Set 29]. Disponível em: http://conselho.saude.gov.br/resolucoes/2011/Reso441.pdf.

de projetos de pesquisa adicionais que auxiliarão no aprimoramento desta vacina ou no desenvolvimento futuro de outras vacinas de COVID-19. O participante poderá retirar sua permissão a qualquer momento, sendo, nesse caso, colocado o material a seu dispor. Este material biológico não será comercializado ou utilizado para a elaboração de produtos comerciais. Todo o material biológico será armazenado e será identificado com o número de identificação recebido pelo participante após a sua inclusão no estudo.

4.4 Descrição dos riscos

Neste estudo, os riscos para os participantes estão associados à punção venosa e *swab* nasal para coleta de material biológico.

4.4.1 Detalhamento dos riscos do estudo

4.4.1.1 Punção venosa

Os riscos associados à punção venosa incluem a necessidade ocasional de mais de uma punção durante a realização da coleta, dor e hematoma no local da punção venosa. A ocorrência de desmaio ou infecção no local da punção são raros. Para minimizar esses riscos, a coleta de sangue será realizada por pessoal treinado, experiente, utilizando material individual, descartável e técnica asséptica de coleta.

De acordo com a Portaria n° 1.353 de 14 de junho de 2011 do Ministério da Saúde, o volume de sangue aceitável para coleta é de nove (9) mL/kg de peso para homens e de oito (8) m/kg de peso para mulheres, sendo que um indivíduo pode realizar uma doação de sangue até três vezes por ano. Portanto, o volume de sangue coletado durante o estudo pode se considerar como seguro para os participantes.

4.4.1.2 *Swab* nasal

Os riscos associados à coleta do *swab* nasal são dor e desconforto no momento da coleta, sangramento nasal no momento da coleta ou logo após, geralmente de pequena magnitude, autolimitados e sem comprometimento clínico relevante.

4.4.2 Descrição dos benefícios antecipados ao participante do estudo

Os participantes não receberão qualquer benefício direto pela participação neste estudo, exceto a proteção contra a infecção por SARS-CoV-2 na população de risco vacinada, que será antecipada ao calendário vacinal proposto pelo PNI e pela Prefeitura de Manaus. Um benefício indireto será o monitoramento trimestral da imunidade do participante.

Durante toda a vigência do estudo, os participantes podem se direcionar a qualquer tempo para realização de testagem gratuita e individualizada (*swab* nasal) para COVID-19, sempre que apresentaram sintomas gripais, quantas vezes forem necessárias. Para isso, disponibilizamos um posto de acolhimento e diagnóstico localizado na Arena Poliesportiva Amadeu Teixeira, que funcionará de segunda à sexta, das 7 às 19h, e aos sábados, das 7 às 13h.

4.4.3 Descrição da relação entre os riscos potenciais e benefícios antecipados

Atualmente, a COVID-19 encontra-se amplamente distribuída, apresenta uma alta incidência no Brasil e os profissionais de segurança pública (policiais) e professores se encontram em risco elevado. Portanto, os esforços para ampliar a vacinação para pessoas com fatores de risco nestas populações representam um grande benefício

4.4.4 Descrição de custos e justificativa de reembolsos ou compensações

A participação no estudo não terá nenhum custo para os participantes. Os participantes não receberão qualquer compensação em dinheiro para participar deste estudo.

4.5 Confidencialidade

Todas as informações relacionadas com o estudo serão armazenadas de forma segura no centro de pesquisa, em arquivos trancados com chave – quando físico – ou em pasta eletrônica com acesso restrito – quando eletrônico. Todas as amostras de laboratório, relatórios, formulários administrativos e para coleta de dados serão identificados apenas pelo número de identificação dos participantes com o objetivo de manter a sua confidencialidade.

Informações que permitam a identificação do participante só serão acessíveis aos membros da equipe de pesquisa encarregados do cuidado do participante, aos auditores e aos inspetores das autoridades regulatórias e éticas dentro do marco da legislação vigente, quando necessário.

4.6 Acesso aos dados

Os dados serão controlados e cuidados pela equipe do Investigador principal, que será responsável por realizar backups periódicos e controlar o acesso dos profissionais a esses dados, especialmente os dados identificáveis. Os dados serão disponibilizados a pedido das autoridades regulatórias dentro do marco legal vigente.

4.6.1 Divulgação dos resultados ao público

A divulgação completa do estudo será realizada através de publicações científicas revisadas por pares. A decisão de autoria das publicações seguirá os Requerimentos Uniformes para Encaminhamento de Manuscritos a Revistas Biomédicas do Comitê Internacional de Editores de Revistas Médicas disponível no site http://www.icmje.org.

Um comitê *ad hoc* com representantes do patrocinador, do fabricante e dos pesquisadores se encarregará de discutir as propostas de divulgação de resultados. Membros desse comitê escreverão o rascunho do manuscrito da publicação e encaminharão para os possíveis coautores para aportes e aprovação. O patrocinador deverá ser consultado com quatro semanas de antecedência do encaminhamento de qualquer resultado derivado deste estudo para divulgação em evento científico

ou publicação. O estudo possui registro em base de dados pública (ClinicalTrials.gov/NTC04789356) desde antes de iniciar o recrutamento e seus resultados serão resumidos nesse mesmo registro. Nenhum resultado do estudo poderá ser divulgado na mídia sem que seja apresentado anteriormente em publicação revisada por pares.

4.6.2 Comunicado aos participantes dos achados do estudo

Será divulgado um comunicado de imprensa com os principais achados relatados nessa publicação científica.

ANEXO A

Escala de progressão clínica de infecção por SARS-CoV-2. Adaptada de proposta da Organização Mundial da Saúde[69].

Descrição	Score
Não infectado, RNA viral não detectado	0
Assintomático, RNA viral detectado	1
Sintomático, independente	2
Sintomático, precisa de ajuda	3
Hospitalizado*, sem precisar de oxigênio	4
Hospitalizado, oxigênio suplementar por máscara ou cânula nasal	5
Hospitalizado, oxigênio por ventilação não invasiva ou de alto fluxo	6
Intubação e ventilação mecânica, $P0_2/FiO_2 \geq 150$ ou $SpO_2/FiO_2 \geq 200$	7
Ventilação mecânica $P0_2/FiO_2 < 150$ ($SpO_2/FiO_2 < 200$) ou vasopressores	8
Ventilação mecânica $P0_2/FiO_2 < 150$ ($SpO_2/FiO_2 < 200$) e vasopressores, dialise ou oxigenação extracorpórea	9
Óbito	10

* Se a hospitalização for unicamente por isolamento, registre o status com paciente ambulatorial

[69] Marshall JC, Murthy S, Diaz J, Adhikari NK, Angus DC, Arabi YM, et al. A minimal common outcome measure set for COVID-19 clinical research. Lancet Infect Dis [Internet]. 2020 Aug;20(8):e192–7.

ANEXO B

Colaboradores	Instituição	Currículo lattes
Marcus Vinícius Guimarães de Lacerda (Investigador Principal)	Fundação de Medicina Tropical Dr. Heitor Vieira Dourado Instituto Leônidas e Maria Deane – FIOCRUZ/AM	http://lattes.cnpq.br/8492376468047417
Maria Paula Gomes Mourão (co-Investigador Principal)	Universidade do Estado do Amazonas Fundação de Medicina Tropical Dr. Heitor Vieira Dourado	http://lattes.cnpq.br/6752341165899077
Wuelton Marcelo Monteiro (Co-investigador)	Fundação de Medicina Tropical Dr. Heitor Vieira Dourado Universidade do Estado do Amazonas	http://lattes.cnpq.br/4986967857234820
Gisely Cardoso de Melo (Co-investigadora)	Fundação de Medicina Tropical Dr. Heitor Vieira Dourado Universidade do Estado do Amazonas	http://lattes.cnpq.br/5566457348830121
Fernando F. De A. E Val (Co-investigador)	Fundação de Medicina Tropical Dr. Heitor Vieira Dourado	http://lattes.cnpq.br/7664318780210160
Vanderson de S. Sampaio (Co-investigador)	Fundação de Medicina Tropical Dr. Heitor Vieira Dourado Fundação de Vigilância em Saúde (FVS-AM)	http://lattes.cnpq.br/0039836167659650
Mariana Simão Xavier (Co-investigadora)	Fundação de Medicina Tropical Dr. Heitor Vieira Dourado	http://lattes.cnpq.br/1896653809437544
Djane Clarys Baia da Silva (Co-investigador)	Instituto Leônidas e Maria Deane – FIOCRUZ/AM	http://lattes.cnpq.br/1446146671158093

José Diego de Brito Sousa (Co-investigador)	Fundação de Medicina Tropical Dr. Heitor Vieira Dourado	http://lattes.cnpq.br/0423198754111442
Felipe Gomes Naveca (Co-investigador)	Instituto Leônidas e Maria Deane – FIOCRUZ/AM	http://lattes.cnpq.br/3396741165569463
Gustavo Adolfo S. Romero (Co-investigador)	Universidade de Brasília	http://lattes.cnpq.br/8497079129889221
Ricardo Palácios (Co-investigadora)	Instituto Butantan	http://lattes.cnpq.br/3860002348093688
Alex Martins (Co-investigador)	Universidade do Estado do Amazonas<br>Fundação de Medicina Tropical Dr. Heitor Vieira Dourado	http://lattes.cnpq.br/3661607448132704
Fabíola Mendonça da Silva Chui (Co-investigadora)	Universidade do Estado do Amazonas<br>Fundação de Medicina Tropical Dr. Heitor Vieira Dourado	http://lattes.cnpq.br/1947773249131833
Maurício Lacerda Nogueira (Co-investigador)	Faculdade de Medicina de São José do Rio Preto	http://lattes.cnpq.br/0565062540207457
Guilherme Rodrigues Fernandes Campos (Co-investigador)	Faculdade de Medicina de São José do Rio Preto	http://lattes.cnpq.br/3664009323158163
Comitê de Monitoramento de Dados e Segurança		
Ivo Müller	Institute Pasteur (Austrália)	https://www.wehi.edu.au/people/ivo-mueller
Quique Bassat Orellana	ICREA/ ISGLOBAL (Espanha)	https://www.isglobal.org/en/researchers/-/profiles/1900
